# Structural and Mechanistic Basis of Zinc Regulation Across the *E. coli* Zur Regulon

**DOI:** 10.1371/journal.pbio.1001987

**Published:** 2014-11-04

**Authors:** Benjamin A. Gilston, Suning Wang, Mason D. Marcus, Mónica A. Canalizo-Hernández, Elden P. Swindell, Yi Xue, Alfonso Mondragón, Thomas V. O'Halloran

**Affiliations:** 1 Department of Chemistry and The Chemistry of Life Processes Institute, Northwestern University, Evanston, Illinois, United States of America; 2 Department of Chemical and Biological Engineering, Northwestern University, Evanston, Illinois, United States of America; 3 Department of Molecular Biosciences, Northwestern University, Evanston, Illinois, United States of America; Rutgers University-Robert Wood Johnson Medical School, United States of America

## Abstract

Commensal microbes, whether they are beneficial or pathogenic, are sensitive to host processes that starve or swamp the prokaryote with large fluctuations in local zinc concentration. To understand how microorganisms coordinate a dynamic response to changes in zinc availability at the molecular level, we evaluated the molecular mechanism of the zinc-sensing zinc uptake regulator (Zur) protein at each of the known Zur-regulated genes in *Escherichia coli*. We solved the structure of zinc-loaded Zur bound to the P*_znuABC_* promoter and show that this metalloregulatory protein represses gene expression by a highly cooperative binding of two adjacent dimers to essentially encircle the core element of each of the Zur-regulated promoters. Cooperativity in these protein-DNA interactions requires a pair of asymmetric salt bridges between Arg52 and Asp49′ that connect otherwise independent dimers. Analysis of the protein-DNA interface led to the discovery of a new member of the Zur-regulon: *pliG*. We demonstrate this gene is directly regulated by Zur in a zinc responsive manner. The *pliG* promoter forms stable complexes with either one or two Zur dimers with significantly less protein-DNA cooperativity than observed at other Zur regulon promoters. Comparison of the *in vitro* Zur-DNA binding affinity at each of four Zur-regulon promoters reveals ca. 10,000-fold variation Zur-DNA binding constants. The degree of Zur repression observed *in vivo* by comparison of transcript copy number in wild-type and Δ*zur* strains parallels this trend spanning a 100-fold difference. We conclude that the number of ferric uptake regulator (Fur)-family dimers that bind within any given promoter varies significantly and that the thermodynamic profile of the Zur-DNA interactions directly correlates with the physiological response at different promoters.

## Abstract 2

Structural, thermodynamic, and gene expression studies provide a comprehensive picture of how the bacterial metalloregulatory transcriptional repressor Zur achieves its exquisite sensitivity to zinc concentrations.

## Author Summary

Zinc is an essential nutrient for most organisms, with the Zn^2+^ ion performing numerous structural, regulatory, and catalytic roles in a range of proteins. However, this nutrient can neither be synthesized nor degraded and individual cells need to be able to maintain steady levels of zinc in the face of near-zero or excessively high environmental concentrations. Here we look at how the bacterium *E. coli* does this, by examining the structure and function of Zur, a transcriptional repressor that is exquisitely sensitive to Zn^2+^ concentration. Although the structures of related Zur proteins on their own are known, here we show how *E. coli* protein binds to DNA and explain its extreme sensitivity and specificity (it responds to Zn^2+^ concentrations in the femtomolar range). Our results reveal how the Zur protein switches on and off a bank of bacterial genes that control zinc physiology. Extensive analysis of protein-DNA interactions revealed both a surprising degree of cooperativity and an extremely large range of Zur-DNA binding affinities across the set of genes known as the Zur regulon. The results provide strong support for a controversial idea that the thermodynamics of an ensemble of protein-DNA interactions play a dominant role in the physiological control of gene regulation networks. In addition, we have used our structural and thermodynamic analysis to identify a novel gene target of Zur regulation.

## Introduction

Zinc fluxes are involved in regulating a wide variety of cellular functions, including host immune activation [1], malaria parasite invasion of erythrocytes [2], oocyte maturation and fertilization [3],[4], glucose-induced insulin secretion [5], as well as the expression of a wide range of microbial genes responsible for metal homeostasis and pathogenicity [6],[7]. It is becoming increasingly apparent that the ability of commensal organisms to adapt to the host environment depends upon the ability to withstand large fluxes in zinc availability that are produced by the host [8]. The mechanisms by which specific factors mediate these dynamic zinc-responsive events are unclear. Cellular quotas, i.e., the number of atoms per cell, for essential transition metals such as zinc are tightly controlled in the face of changing metal concentrations in the surrounding growth environment [9],[10]. Zinc is an important factor in understanding colonization by both beneficial enteric bacterial species as well as infection by pathogenic microorganisms. In each case, the host can trigger rapid changes in zinc availability leading to either starvation or saturating conditions to alter the local bacterial environment. Microbes use a diverse set of metal-specific sensors known as metalloregulatory proteins to respond to changes in metal concentration in the immediate environment [7],[11],[12]. These transcription factors control expression of many diverse factors including membrane bound metal ion transporters that optimize cellular physiology in the face of dynamic shifts in metal availability.

Zinc uptake regulator (Zur) is a homolog of one of the first metalloregulatory proteins identified, namely the ferric uptake regulator protein (Fur). Fur has been shown to regulate over ninety genes in *Escherichia coli* in response to changes from growth in iron deplete to iron replete conditions [13]. Zur regulons have been identified in a diverse range of organisms such as *E. coli* [14], *Bacillus subtilis* [15], *Listeria monocytogenes* [16], *Staphylococcus aureus* [17], *Mycobacterium tuberculosis* [18], *Yersinia pestis* [19], *Corynebacterium glutamicum* [20], and *Pseudomonas aeruginosa* [21], to name a few. Like Fur, Zur proteins regulate the expression of a number of genes that play a role in virulence of a pathogenic organism, including the ATP-dependent ZnuABC zinc transport proteins in the commensal gram-negative human pathogen *Neisseria meningitides* [22]. In *E. coli* K-12, the Zur regulon includes the *znuABC* zinc uptake gene cluster in addition to genes encoding a pair of ribosomal proteins (*L31p* and *L36p*) as well as a periplasmic zinc trafficking protein (*zinT*) [23]–[25]. These three cellular processes: zinc importer systems, ribosomal proteins, and periplasmic scavenging proteins remain the only consistent set of Zur regulated genes across bacterial species.

Despite 13 crystal structures of Fur family proteins, neither the atomic structure of a Fur family protein-DNA complex nor the molecular mechanism by which metal binding in the receptor site triggers changes in DNA-binding are known. To date, the structures of several Fur family members, including the zinc response regulator, Zur, are known in the absence of DNA and the nature of the core DNA element recognized by these proteins remains unclear. DNase I footprinting and thermodynamic studies of the metal affinity of *E. coli* Zur have demonstrated that the DNA binding activity of Zur is highly selective to changes in Zn^2+^ ion concentrations over other transition metals and that *E. coli* Zur switches off transcription with a K_d_ for Zn^2+^ in the sub-femtomolar range, corresponding to less than one chelatable atom of zinc per cell [26]. This ultrasensitivity of Zur to changes in zinc concentration suggests that bacteria are intolerant of free zinc in the cytosol under normal growth conditions. Intriguingly, this small protein (19 kDa) protects an unexpectedly large region (∼30 bp) of the *znuABC* (P*_znuABC_*), the *L31p* (P*_L31p_*), and *zinT* (P*_zinT_*) operators under Zn^2+^-saturating conditions [14]. Given that *E. coli* accrue zinc and iron to similar levels under most growth conditions, the small number of Zur regulated genes stands in contrast to the copious number of genes regulated by *E. coli* Fur. Here we uncover the molecular basis for the specific transcriptional responses at the structural and thermodynamic level. Our structure determination of the *E. coli* Zur protein bound to the *znuABC* operator combined with thermodynamic and cooperativity analyses of Zur-binding at all of the known Zur-regulated promoters lead to a comprehensive view of differential expression patterns in this regulon. Studies of other known repressor complexes suggest that protein-DNA cooperativity could be mediated through DNA distortion [27]–[29]. However, here we find that the molecular basis of Zur-DNA cooperativity arises from communication between two dimers bound on adjacent faces of the DNA duplex through a pair of salt bridges. The structural and thermodynamic data allow identification of the specific-sequence recognition elements that give rise to differential DNA recognition within the Zur family of proteins. Finally, we demonstrate the predictive value of this combined structure/thermodynamic/bioinformatic approach by identifying a new Zur-regulated gene, *pliG*. These results open the door to the identification of a larger Zur regulon and underscore the idea that cooperativity in a biological sensor-analyte system is not simply based on the affinity of the receptor for the Zn^2+^ ion, it also depends upon downstream interactions and activities of the transcriptional machinery.

## Results

### Overall Fold and Characterization of Zinc Binding Sites

**Figure 1:**
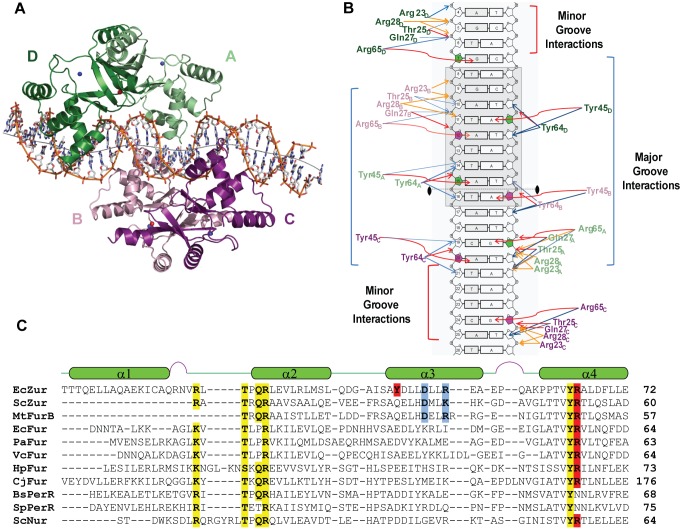
Structure of (Zur_2_)_2_-DNA complex and specific interactions with DNA. (A) Overall structure of Zn-Zur-33mer DNA complex. The four protein subunits are labeled A–D: dimer 1 contains monomers A and D (green); dimer 2 consists of chains B and C (purple). The DNA axis was generated by Curves+ [58] and is shown in grey. (B) 2D representation of Zur-P*_znuABC_* promoter contacts. Amino acid residues of Zur contacting the DNA are colored by dimer, green for dimer 1 and purple for dimer 2. The subscript of each amino acid refers to the monomer chain involved in binding. The extended −10 RNA polymerase binding site is portrayed in a grey outline. The 2-fold axis is shown between bases 15 and 16. Hydrogen bonds between protein and DNA are shown in red. Hydrophobic interactions are shown in blue and lastly electrostatic interactions are shown in orange. Interactions were obtained with the program Monster [92]. (C) Structure based alignment of EcZur and the known structures of the Fur family. The secondary structure elements of the Zur crystal structure are shown above the corresponding sequence of the Fur proteins. Highlighted in yellow are conserved DNA-binding residues. Highlighted in blue are the conserved cooperativity linker salt bridge residues. Highlighted in red are Tyr45 and the conserved Arg65, which make hydrogen bonds to the DNA bases.

The structure of *E. coli* Zur (EcZur) protein in complex with a 31 bp duplex derived from the *znuABC* operator (P*_znuABC_*) was determined by X-ray crystallography using multiwavelength anomalous dispersion (MAD) data collected at both high energy and the zinc absorption edge and refined to 2.50 Å (Table 1). Each EcZur monomer includes two domains: an N-terminal DNA-binding domain and a C-terminal dimerization domain. The DNA-binding domain (α2–α4) contains a helix-turn-helix (HTH) motif and is connected to the C-terminal domain (α5/β5) using a characteristic winged-helix motif. The three dimensional model of the complex shows the central portion of the operator DNA surrounded by four Zur monomers forming a dimer of dimers. Analysis of the complex reveals the HTH motif interacts with the major and minor grooves of the DNA. Figure 1 shows the final model which includes amino acid residues 5–152 of four protein monomer chains (designated chains A, B, C, D), eight zinc ions, and two 31 bp strands of DNA (designated chains Y and Z) in the asymmetric unit. The nature of the dimer of dimers ((Zur_2_)_2_) structure indicated that there were two possible orientations for the DNA to adopt in the crystal. Using DNA brominated at asymmetric points indicated that the crystal has an equal mixture of both DNA orientations. Given the pseudo-palindromic nature of the DNA sequence none of the protein-DNA contact sites were affected by this lack of directionality; however, several bases represent an average over the two conformations. For clarity in the discussion only one DNA orientation is considered.

**Table 1:** Data collection and refinement statistics.

Data Collection and Refinement	Statistic	Subcategory		Zn-Zur+**31mer2bpOH** (Native)	Zn-Zur+**31mer2bpOH** (Zinc Anomalous Data)
**Data collection**		**Space group**		C2	C2
	**Cell dimensions**	***a*** **, ** ***b*** **, ** ***c*** ** (Å)**		193.4, 80.5, 98.8	194.9, 80.7, 99.4
		α, **β, γ (°)**		90.0, 120.2, 90.0	90.0, 120.5, 90.0
		**Resolution (Å)**		36.4–2.50 (2.61–2.50)^a^	36.5–2.51 (2.62–2.51)
		**Wavelength (Å)**		0.97872	1.2782
		***R*** **_merge_**		0.046 (0.29)	0.045 (0.54)
		**R_measure_**		0.054 (0.331)	0.067 (0.725)
		***I*** **/σ(** ***I*** **)** b		19.4 (5.0)	17.5 (2.5)
		**Completeness (%)** c		99.8 (99)	98.4 (95.3)
		**Multiplicity** d		4.1 (4.2)	4.4 (4.3)
	**Number of reflections**	**Total**		188,485 (22,794)	197,429 (22,915)
		**Unique**		45,429 (5,460)	44,954 (5,281)
**Phasing**		**MFID** e			0.092
		**Number of sites** **Phasing Power** f		8	8
			**Dispersive (centric/acentric)**		0.513/0.619
			**Anomalous(acentric) R-Cullis**	0.528	
			**Isomorphous(cen/acen)**		0.89/0.905
			**Anomalous**	0.944	0.918
		**Figure of Merit (centric/acentric)**			0.2624/0.1663
**Refinement**		**Resolution (Å)**		36.4–2.50 (2.53–2.5)	
	**Number of reflections**	**Working**		45,422 (2,742)	
		**Test**		4,461 (110)	
		***R*** **_work_**		0.2175 (0.3066)	
		***R*** **_free_**		0.2569 (0.3250)	
	**Number of Atoms**	**Protein/DNA**		6,618	
		**Ligand/ion**		8	
	***B*** **-factors (Å^2^)**	**Protein**		61.9	
		**DNA**		64.3	
		**Zn**		59.6	
	**R.m.s. deviations**	**Bond lengths (Å)**		0.0095	
		**Bond angles (°)**		0.953	
	**Ramachandran Plot** g	**Favored**		95.6%	
		**Allowed**		4.06%	

Each dataset was collected from a single crystal.

a Values in parentheses are for highest-resolution shell.

b Mean I/σI as defined by Scala [93].

c Percentage of completeness and anomalous completeness as defined by Scala [93].

d Multiplicity and anomalous multiplicity as defined by Scala [93].

e Mean fractional isomorphous difference (MFID) = Σ ∥*F*1|−|*F*2∥/Σ |*F*1|, where |*F*1| = reference structure factor amplitude and |*F*2| = compared structure factor amplitude.

f Phasing power = r.m.s. (|*F*h|/E) where |*F*h| = heavy atom structure factor amplitude and E = residual lack of closure error, reported for all acentric reflections.

g Values from Molprobity [88].

**Figure 2:**
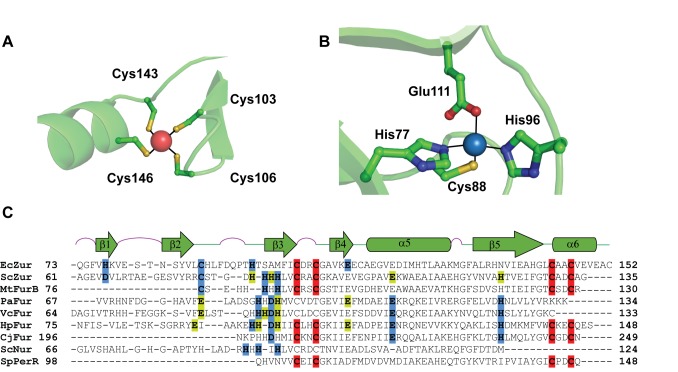
Zinc coordination environments used by *E. coli* Zur. (A) Site A ligand coordination for the sulfur-rich zinc site (Zn shown in red). (B) Site B ligand coordination for the nitrogen/oxygen-rich zinc site (Zn shown in blue). (C) Structure based alignment highlighting the zinc-coordinating amino acid side chains in known Zur and Fur structures. The sulfur-rich, nitrogen/oxygen-rich, and so-called “third zinc” binding site ligands are shown in red, blue, and yellow, respectively. Note the third zinc binding site is observed in less than half of the structurally characterized family members. This alignment shows a high degree of conservation of the tight-binding sulfur-rich zinc site, while the other two zinc binding sites vary significantly amongst known Fur family structures.

Anomalous difference data collected at the Zn edge reveal two distinct Zn^2+^ binding sites in each of the four Zur monomers: the Zn^2+^ in site A is bound to four sulfur atoms from Cys103, Cys106, Cys143, and Cys146 and the Zn^2+^ in site B is bound by residues His77, Cys88, His96, and Glu111 (Figure 2). These results are consistent with earlier extended X-ray absorption fine structure (EXAFS) experiments demonstrating the presence of two Zn-binding sites per monomer: a tighter binding site that is dominated by sulfur coordination and a weaker site that is richer in nitrogen/oxygen atoms [30]. Intriguingly, neither the site A nor the site B zinc coordination in EcZur is observed in other structurally characterized members of the Zur regulon (i.e., ZnuA, L31p, and ZinT) [31]–[37].

**Figure 3:**
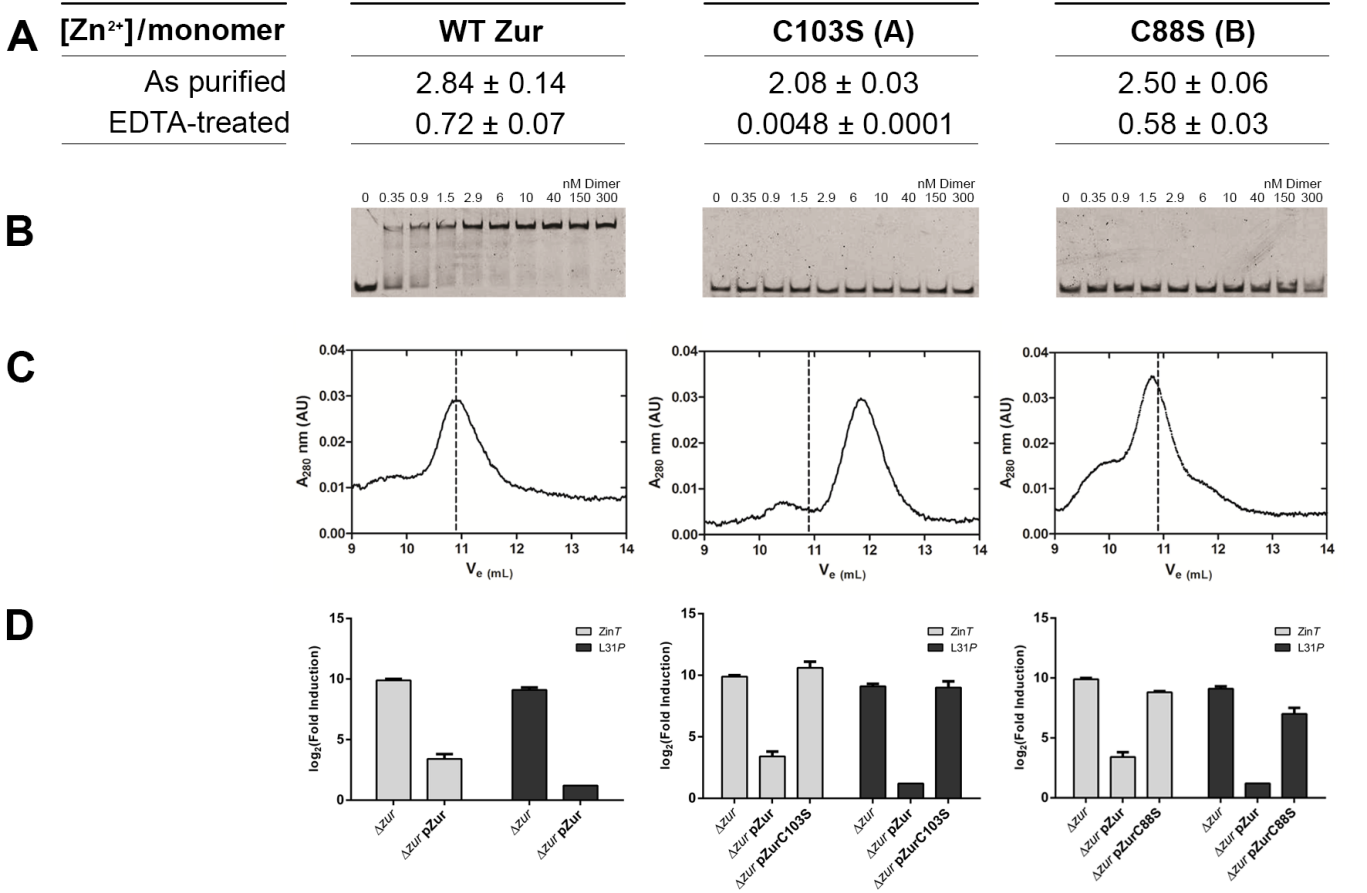
Characterization of WTZur Zn-binding with A-site (C103S) and B-site (C88S) mutant. (A) Metal contents of Zur measured by inductively coupled plasma mass spectrometry (ICP-MS). Analysis of both purified and EDTA-treated proteins were measured in triplicate. (B) DNA binding activity of WT and mutant Zur proteins analyzed by EMSA gel shifts of the *znuABC* operator. Using these qualitative gel shift experiments it is apparent that a single site-directed mutation in site A or site B have a dramatic effect on DNA-binding affinity. (C) Analytical gel filtration chromatograms of WT Zur, site A mutant C103S, and site B mutant C88S. The dotted lines in (B) and (C) indicate the position of the elution volume (V_e_) of WT protein as a reference (10.9 ml). These experiments demonstrate that site A residues are critical for Zur dimerization. (D) *In vivo* complementation assay measurement of L31p and zinT expression demonstrate that mutating either site A or site B removes the ability of Zur to repress transcription. See Data S1 for the raw data used to generate each panel.

Structure-based alignment of *E. coli* Zur with sequences of structurally characterized Fur-family proteins shows some conservation of zinc-binding residues. The most highly conserved of these are the two C-X-X-C motifs of the sulfur-rich site A (Figure 2C). Amongst the Zur sub-family members C88 is the most conserved residue in the B site (note: MtFurB is a Zur protein that controls regulation of zinc uptake genes [38]). To test whether zinc occupancy in these two sites is important for repressor function of EcZur, we mutated a conserved residue in each site to serine and compared the ability of these variants to compliment Zur activity in the zur-null strain. Mutations in either site A (C103S) or site B (C88S) lead to a complete loss of Zur-regulated transcription *in viv*o (Figure 3). Both mutant proteins were stably expressed, isolated, and evaluated in metal-binding, DNA-binding, and dimerization assays. Analysis of zinc stoichiometry by inductively coupled plasma mass spectrometry (ICP-MS) reveals that these mutant proteins can be loaded in the presence of excess zinc, but to a lesser extent than wild-type Zur (WTZur) (Figure 3). To provide a rigorous test of zinc binding activity, i.e., one that mimics better conditions inside the cell, we dialyzed zinc-loaded proteins against a stringent Zn^2+^-chelating buffer solution that contains 25 mM EDTA. Under these zinc-limiting conditions, the site A mutant does not bind Zn^2+^ to any significant degree, whereas the site B mutant binds zinc to a similar extent as WTZur (0.5–0.7 zinc/monomer). To test whether the DNA binding activity of WTZur requires Zn^2+^ occupancy at these sites, we titrated these zinc-loaded Zur proteins in native gel electrophoretic mobility shift assays. We find saturation of DNA binding at ca. 3 nM dimer for wild-type (WT) protein. However, neither the site A (C103S) nor the site B (C88S) mutant exhibits any DNA binding activity at the highest protein concentrations examined (Figure 3B). When these assays are repeated in the presence of excess Zn^2+^ a significant weakening of affinity is still observed (Figure S1). Thus DNA binding activity of EcZur is severely limited when either of the zinc binding sites is compromised. Intriguingly we find that the site A mutant protein does not form a stable dimer, whereas the site B mutant does so under these conditions (Figure 3C). These findings underscore the idea that Zn^2+^ occupancy of both site A and site B is important for inducing the DNA binding conformation of Zur and furthermore that Zn^2+^ occupancy of site A can influence dimer formation. These results for EcZur corroborate studies of the role of analogous zinc binding sites in Zur proteins from *S. coelicolor* (ScZur) and *B. subtilis* (BsZur) [39],[40]. Intriguingly, the EcZur site A Zn^2+^ ions superimpose well with both open and closed structures of other dimer Fur proteins (Table 2), whereas the site B zinc ions do not. These similarities in the A site and the differences in the B site provide a structure-based perspective for how zinc occupancy of each sites leads to stabilization of the high-affinity DNA binding conformation of Zur. For example, zinc occupancy at site A involves binding to two C-X-X-C motifs that directly flank the dimerization domain (α5/β5) and these serve to stabilize the dimer interface. The site B zinc binding pocket contains β-strands β1–β3, and is linked by a short 4 amino acid loop to one of two of the principal DNA-recognition helixes, namely α4. We anticipate that zinc occupancy of this site adds significantly to the stabilization of the closed DNA-binding conformation of Zur.

**Table 2:** Superposition of *E. coli* Zur with known structures of Fur family members.

Crystal Structure	Superpose RMSD (Å)	RAPIDO RMSD (Å)
**Closed-form**		
*E. coli* Fur	1.0	1.9
*S. coelicolor* Zur	1.2	2.3
*S. coelicolor* Nur	1.3	4.1
*B. subtilis* PerR-Zn-Mn	1.4	3.6
*V. cholera* Fur	1.6	4.0
*P. aeruginosa* Fur	1.7	4.6
**Open-form**		
*B. subtilis* PerR-Ox	2.9	14
*M. tuberculosis* FurB/Zur	3.0	11
*H. pylori* Fur	3.1	5.3
*B. subtilis* Apo-PerR-Zn	3.1	14
*C. jejuni* Fur	4.3	14
*S. pyogenes* PerR-Ni-Zn	4.5	15
*S. pyogenes* PerR-Zn	4.7	15

Superposition of each known structure with the protein dimers in the Zur-DNA structure was performed using the programs Superpose [52] and RAPIDO [94]. Reported here at the global superposition values for the automatically selected α-carbon structure based alignment. This analysis demonstrates that the known structures with low rmsd values correspond to protein structures in the “closed” state, capable of binding to their respective operator DNA.

Nearly half of the 13 different crystal structures of Fur family members determined in the absence of DNA are found in the open conformation and the remaining structures are described as closed. When compared to EcZur bound to DNA, both the open and closed conformations show good agreement across the C-terminal dimerization domains, but differ significantly in the relative positioning of the N-terminal DNA binding domains. The open conformation (*B. subtilis* Apo-PerR-Zn [41], *B. subtilis*. PerR-Oxo [42], *M. tuberculosis* FurB [38], *Helicobacter pylori* Fur [43], *Streptococcus pyogenes* PerR [44],[45], and *Campylobacter jejuni* Fur [46]) has been postulated to have a low affinity for DNA, as the DNA-binding domains are too far apart to interact with a DNA molecule. Structures identified in the closed conformation (*Streptomyces coelicolor* Zur [40], *B. subtilis* PerR-Zn-Mn [47], *S. coelicoler* Nur [48], *E. coli* Fur [49], *Vibrio cholera* Fur [50], and *P. aeruginosa* Fur [51]) have been proposed to both correspond to the fully metal loaded form of the repressor and have a high affinity for DNA. Use of the structural alignment program SuperPose [52] indicates that superposition of the *E. coli* Zur dimers with the known Fur proteins gives the strongest agreement (<1.6 Å rmsd for the α-carbons) with the closed crystal structures. Superpositions with the open form of the Fur proteins show significantly poorer agreement (>3.0 Å rmsd) (Table 2), particularly in the orientation of the DNA-binding domains. The Zur-DNA complex provides the first structural evidence that the previously identified closed conformations of the Fur proteins correspond to the conformation capable of binding DNA, and further reveals the molecular basis of protein-DNA recognition in the unusually long operator sequence.

### Tyr45 and Arg65 Interactions with Zur-Box Purines Define the Recognition Motif

Over 100 contacts between protein and nucleic acid atoms are observed in the (Zur_2_)_2_-DNA complex, and the majority of these are between conserved amino acid side chains and the phosphate backbone. Interestingly, each of the four monomers of *E. coli* Zur makes two hydrogen bond contacts to specific purine DNA bases for a total of eight direct interactions with the P*_znuABC_* operator (Figure 1B). The majority of these contacts are in the major groove and only two types of functional groups are involved in hydrogen bonding. Each monomer A through D has two hydrogen bond donors, Tyr45 and Arg65, that interact with the N7 nitrogen of bases G7, A11′, A12, A15, A16′, G19′, A20, G24′ (where ′ denotes non-coding strand) (Figures 1A, 1B, and S2). Conservation of Arg65 among both Zur and Fur family members along with previous mutational analysis suggested that this amino acid is critical for binding to DNA by many proteins of the Fur family [53]. Unexpectedly we find that each monomer uses Tyr45 in addition to Arg65 in direct readout of the DNA sequence element or ‘Zur box.’ Tyr45 is not conserved across all Fur homologs, but is unique to gram negative Zur protein-DNA recognition, and likely provides discrimination between Zur- and Fur-regulated promoters (Table S1). Each of the four monomers recognizes two purines on opposite strands of DNA separated by three base pairs in an R-N-N-N-Y motif. In this motif Arg65 hydrogen bonds to the first purine (most frequently a G) followed by Tyr45 hydrogen bonding to the purine compliment of residue Y on the opposite strand (Figure 1B). In addition to these base specific interactions, the backbone shape or indirect readout is also observed between each Zur monomer and the dozens of electrostatic and hydrophobic contacts with the DNA backbone. We first examined whether binding of both dimers to DNA is required to establish a stable repressor complex in solution in order to understand the key elements within our Zur-DNA recognition and thus provide the best molecular definition of a Zur box. We further examined the solution data to evaluate contributions of several of the contacted bases to the thermodynamics of specific protein-DNA complex formation.

### Zur-DNA Interaction Is Highly Cooperative at the P*_znuABC_* Operator

**Figure 4:**
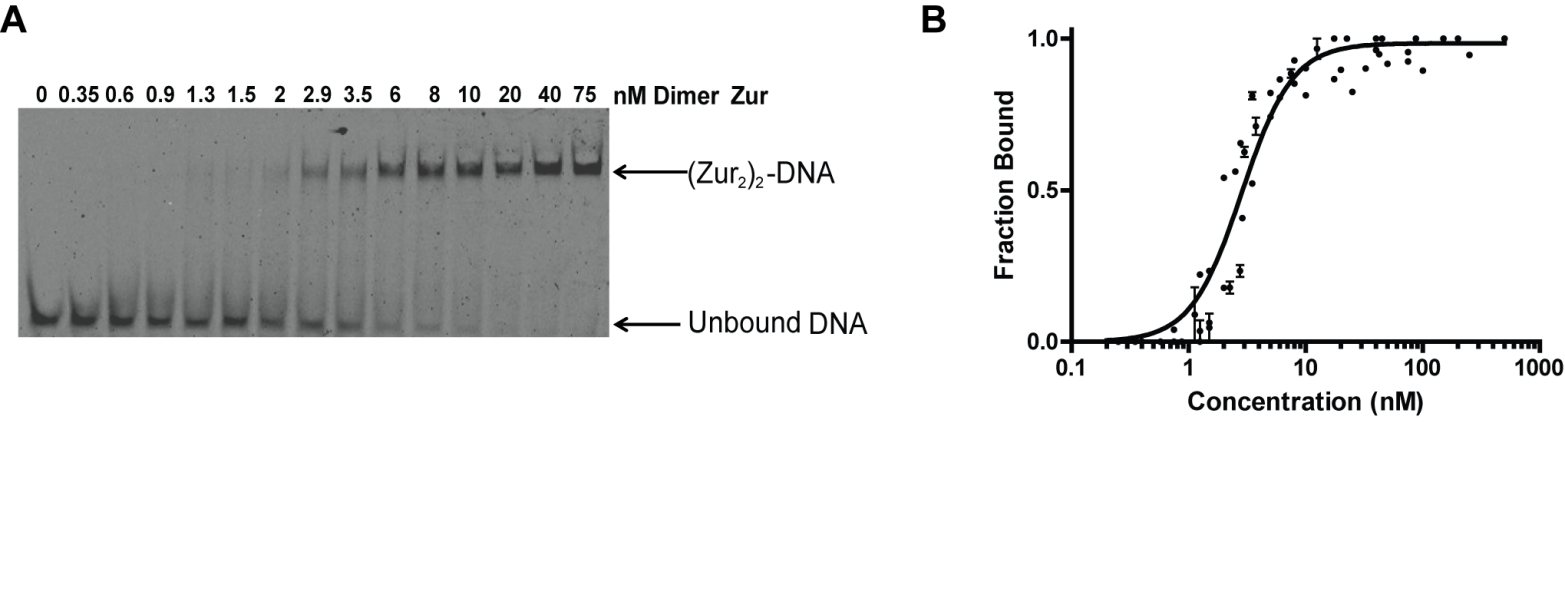
Affinity determination of WT Zur titrations of P*_znuABC_* by EMSA. (A) Representative gel of the Zur affinity for the *znuABC* promoter. A Cy5 labeled DNA fluorescent probe was used to monitor the formation of a DNA-protein complex. Each lane represents a different reaction between protein and DNA, where the DNA and Zn^2+^ concentrations are kept constant (≤45 pM and 50 µM, respectively) as increasing concentrations of protein are added to the sample. The mobility of the shifted species corresponds to an apparent molecular weight of 110 kDa, which corresponds to (Zur_2_)_2_-DNA (see Figure S3). Note the absence of any bands corresponding to the single dimer Zur_2_-DNA intermediate species. (B) Graphical representation of the percentage of bound DNA versus the concentration of Zur protein. The data points presented in this graph are representative of three separate gel shift experiments. A binding isotherm fit to Equation 2b gives a protein-DNA dissociation constant of K_d-app_ = 8.2 (±0.7)×10^−18^ M^2^. Hill plots identify a Hill coefficient of α_H_≥2.0 indicating that protein-DNA binding is highly cooperative (see Figure S4 and Data S2 for the raw data used to generate each plot.).

Native gel shift experiments were used to evaluate binding of the Zur dimer to the *znuABC* promoter in solution. Titrations using samples of WTZur consistently reveal a highly cooperative binding that results in complexes that have two dimers bound to DNA under saturating conditions (Figure 4A). Using the method developed by Orchard and May [54] we find that the protein-DNA stoichiometry in solution is the same as that observed in the crystal structure, namely two protein dimers to one duplex DNA (Figure S3). The absence of an intermediate complex (i.e., one dimer per DNA) coupled with Hill coefficients that are significantly greater than unity (i.e., α_H_>1) (Figure S4) indicate that formation of the dimer of dimers complex is a highly cooperative process. Therefore, the binding of *E. coli* Zur and DNA can be described as a two-step mechanism shown in scheme 1a and 1b:
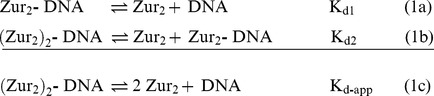
Given the highly cooperative binding of two dimers to the operator, it is not possible to determine microscopic site constants K_d1_ and K_d2_ for each dimer (reactions 1a and 1b or Equation 2a); however, the apparent macroscopic dissociation constant (K_d-app_) for reaction 1c is readily determined by fitting the fraction of DNA bound as a function of total Zur dimer concentration using Equation 2b [55] (Scheme S1):
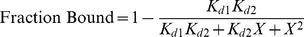
(2a)

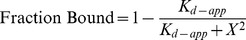
(2b)
Equation 2b is identical to the derived thermodynamic expression used to model a highly cooperative two-to-one equilibrium [56] such as the binding of diphtheria toxin repressor (DtxR) to DNA [29]. Fitting the Zur-DNA titration data with equation 2b (Figure 4) yields an apparent macroscopic dissociation constant K_d-app_ for the cooperative binding of Zur to the *znuABC* promoter of 8.2 (±0.7)×10^−18^ M^2^.

### An Asymmetric Pair of Salt Bridges Is Critical for the Cooperative Binding of Two Dimers to DNA

**Figure 5:**
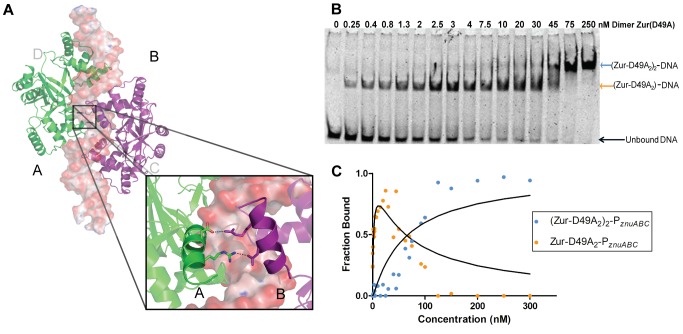
Identification of cooperativity linker and effect on protein-DNA binding. (A) Salt bridge formation between monomers A and B. The image illustrates the communication between the A and B monomers across the dimer-dimer interface. The equivalent interaction is not formed in the other dimer-dimer interface (not shown) (B) Native gel shifts demonstrate the isolation of a single dimer-DNA intermediate in mutant protein D49A unseen in the WT Zur gel shifts. Shown here is a representative gel-shift for Zur(D49A)_2_ titration of P*_znuABC_*. (C) Two-site binding isotherms modeled for the equilibrium for Zur(D49A)_2_ binding corresponding to K_d1_ = 2.1 nM (orange) and K_d2_ = 65 nM (blue). See Data S3 for the raw data used to generate each plot.

We next addressed whether the two dimers communicate with each other through direct contacts or through modulation of local DNA structure. The structure of the (Zur_2_)_2_-DNA complex reveals a subtle but direct communication from one dimer to the other. Two salt bridges between adjacent dimers were observed, between Asp49 from monomer A to Arg52 of monomer B, and vice versa (Figure 5A). Mutant forms of Zur where the salt bridge is removed (D49A or R52A) retain both zinc binding (2 mol zinc per mol Zur monomer) and tight DNA binding activity, but do not exhibit the highly cooperative binding observed for the WT protein. Instead, gel shift assays reveal an intermediary species that is not seen in assays using the WT protein and this species persists throughout the central portion of the titration (Figure 5B). Using gels with varying acrylamide percentages, it was determined that the intermediary protein-DNA species has a stoichiometry of one Zur dimer to one DNA molecule (Figure S3) [57]. In this case, the stepwise binding constants K_d1_ and K_d2_ can be simultaneously estimated by fitting analytical forms of the equilibrium expressions 1a and 1b to the DNA binding data (Equation 2a; Scheme S1). We find the binding of one mutant dimer to the P*_znuABC_* promoter site is significantly favored (K_d1_ = 2.1–2.6 nM) over the binding of a second mutant dimer to an adjacent site (K_d2_ = 65–220 nM) (Figures 5C and S5). The product of these microscopic dissociation constants estimate that the macroscopic K_d-app_ for the overall binding is between 140 and 570×10^−18^ M^2^. When compared to WT protein, the free energy penalty (ΔΔG) for mutating either salt bridge linker is ∼2 kcal mol^−1^. Based on these findings, we conclude that the cooperativity observed in binding of the WT dimers to the DNA surface arises from the two salt bridge contacts between the dimers. Interestingly, the amino acid residues that form this pair of salt bridges are highly conserved within the gram-negative subset of Zur proteins (Table S1), but not in other members of the Fur family. We propose that these pairs of salt bridges act as a “cooperativity linker” and play a central role in the physiology of Zur repression.

### Central Purines Are Key to Dimer-Dimer Binding in Zur Regulon Promoters

**Figure 6:**
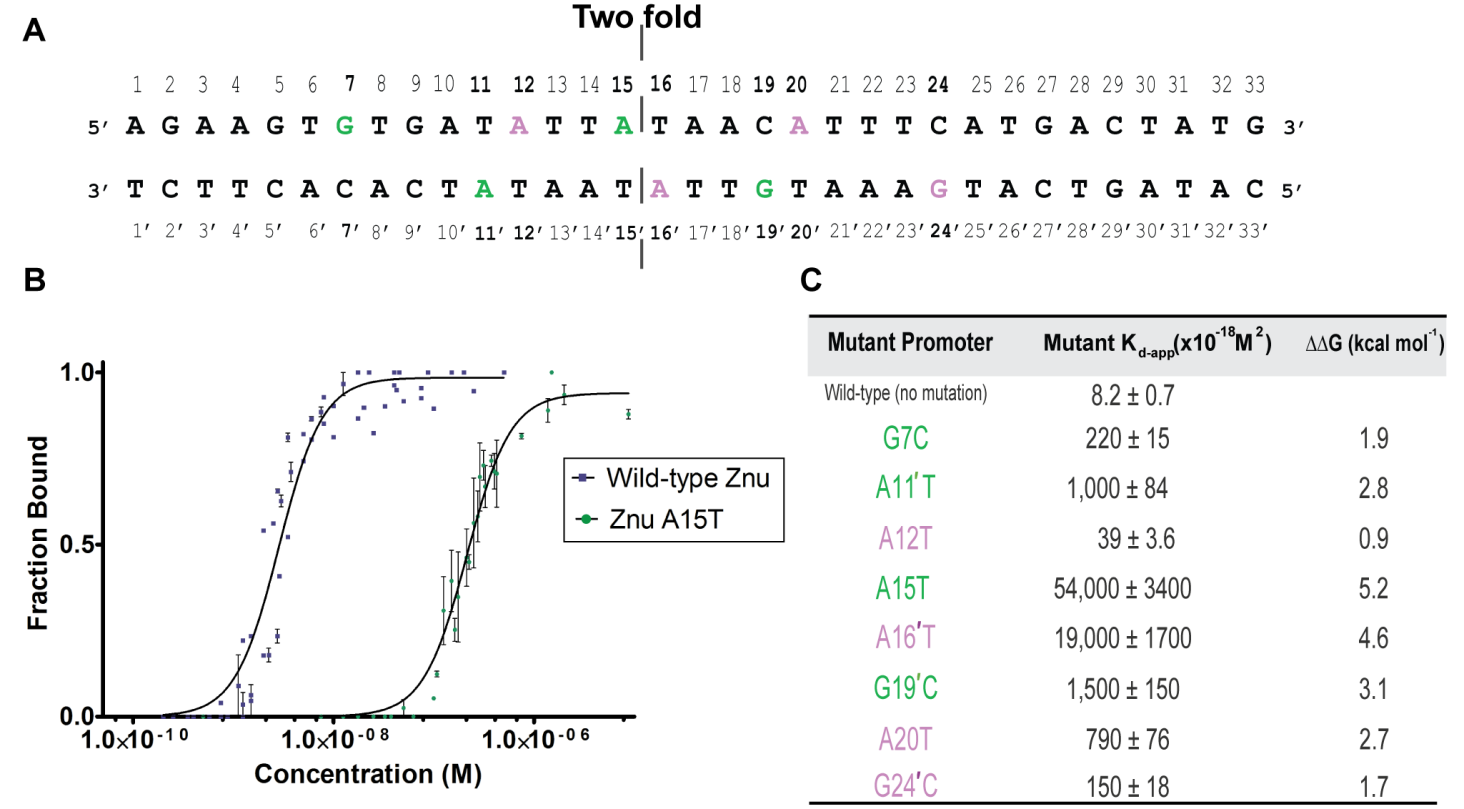
Affinity determinations of *znuABC* purine mutations by EMSA. (A) Sequence of the *znuABC* with corresponding purines of Dimer 1 recognition (green) and Dimer 2 (purple) recognition sites. (B) Representative binding isotherms between the WT *znu* promoter and a single mutation, in this case A15T in the center of the DNA sequence, highlighting the difference in binding affinity. (C) Table summarizes the effect of mutating each purine individually and the relative weakening on the Zur-DNA affinity. In all cases Hill plots indicate that DNA-binding occurred in a cooperative manner α_H_≥1 (See Data S4 for the raw data used to generate each panel.).

In order to elucidate the relative importance of specific Zur/DNA contacts in the Zur-*znuABC* structure, every base involved in hydrogen-bond interactions with Zur was mutated and the affinity of each individual mutant operator DNA was analyzed by titration with WT protein (Figure 6). In all cases, the protein-DNA binding was cooperative; no intermediate species were observed (i.e., corresponding to one Zur dimer per DNA), and the overall DNA-binding affinity was weakened. The thermodynamic analysis of the protein-mutant DNA binding reveals that the central-most bases contacted by Zur, namely A15 and A16′ have the most significant effect on stability of the (Zur_2_)_2_-DNA complex (Figure 6). The importance of the inner most bases relative to the outer bases has been observed previously, and now has structural support [22]. In the Zur-*znuABC* structure, the majority of deviations from ideal B-form DNA behavior occur at the contacted central bases A15 and A16′. Whereas the overall shape of the crystallized DNA is not greatly distorted from ideal B-form behavior (the bend of the *znuABC* DNA is ∼15°), two of the largest major groove widths occur at bases 15 (18.3 Å) and 16 (16.8 Å), significantly larger than 11.4 Å for B-form DNA (Figure S6). In order to accomplish this major groove expansion there must be significant overall unwinding of the DNA. This unwinding is readily quantified using the Curves+ program twist measurements [58], which calculated the *znuABC* DNA twist as 32.6°, nearly 4° smaller than in ideal DNA. In addition, the analysis indicates that the center of *znuABC* DNA has negative roll angles and narrow minor grooves widths of 2.5 Å (base 15) and 2.8 Å (base 20), both significantly narrower than for B-form DNA (5.9 Å). This observation that negative roll angles precede narrow minor grooves has been seen in prior analysis of protein-DNA complexes [59]. However, unlike other protein-DNA complexes, the Zur-DNA structure unwinding occurs at the center of the DNA. Closer examination of the Zur-DNA structure reveals that the inner bases (TATA) are contacted by Tyr45. This inner TATA sequence is unique to *E. coli* Zur and not present in the consensus *E. coli* Fur box [60]. We speculate that the Tyr45 forms a unique set of bonds with the central TATA nucleotides to provide the key difference between *E. coli* Zur/Fur recognition.

### Pattern of Contacts in the (Zur_2_)_2_-P*_znuABC_* Structure Lead to a Novel Zur Regulated Gene

**Figure 7:**
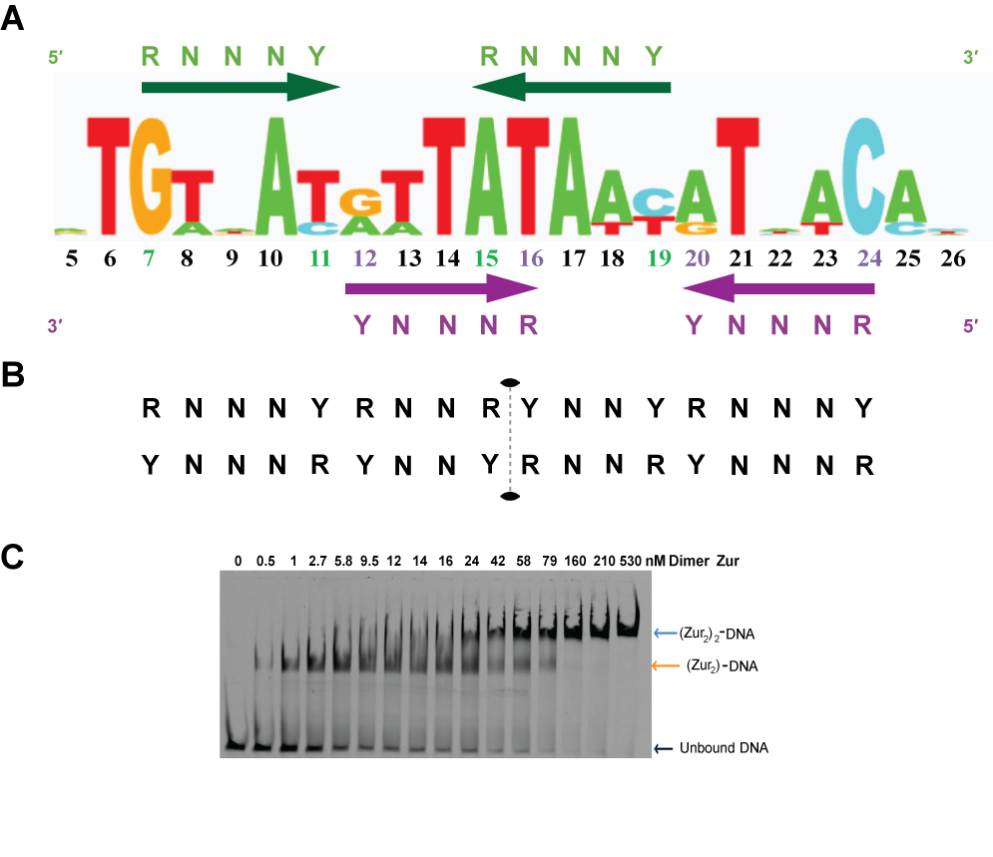
Structure-based pattern for DNA recognition by *E. coli* Zur dimers. (A) Sequence logo representation of the template strand in Zur-DNA recognition based on the four known Zur operators (see text for details). Each number corresponds to the base number in the Zur-DNA crystal structure. The bases in the motif recognized by dimer 1 are highlighted in green, while the dimer 2 recognized bases are highlighted in purple. The purine-N-N-N-pyrimidine (i.e., R-N-N-N-Y) motif is conserved within all four of the operators regulated by Zur. Overlap of the green and purple recognition motifs at positions 15 and 16 highlight the importance of the central AT bases. (B) Purine and pyrimidine pattern of the two dimer DNA recognition of *E. coli* Zur. The sequence dyad is shown with dotted lines. (C) Representative gel for the Zur titration of P*_pliG_*. The presence of the single dimer intermediate provides the ability to calculate individual macroscopic binding constants. For Zur-*PliG* binding isotherms see Figure S6.

Using a structure-based molecular recognition analysis of the (Zur_2_)_2_-P*_znuABC_* complex, we asked if the two other promoters shown to be regulated by *E. coli* Zur (P*_zinT_* and P*_L31p_* [25]) accommodate the same dimer of dimers pattern of base-specific readout. Thermodynamic analysis using gel-shift assays of WT Zur binding to each of these promoters reveals that all three bind Zur in a highly cooperative “all-or-none” manner to form a dimer of dimer complex with DNA. Furthermore, each promoter has multiple interrupted purine-N-N-N-pyrimidine binding motifs, which are recognized by the Zur monomers in the structurally characterized complex with P*_znuABC_* DNA. Comparison of these three sequences allows us to test the elements necessary for establishing an energetically validated Zur box. In each promoter we find two purine-N-N-N-pyrimidine motifs separated by three residues to give RNNNYxxxRNNNY, which serves as the core recognition element for one Zur dimer. The binding of two dimers at overlapping RNNNY sites seen in the structure of the ZnuABC gives rise to a skeletal structure-based dimer-dimer recognition motif: RNNNYRNNRYNNYRNNNY. A summary of this is graphically depicted in terms of colored arrows in Figure 7A where we have superimposed the base pair occurrence in the three known promoters in a sequence walker format. A more specific pattern for a two dimer recognition site for Zur can be described using IUPAC code: TGWNAYRWTATAWYRTNWCA. When this structure based *E. coli* Zur box is compared with the Zur box from other organisms it is clear that the central portion of the sequence is key to the recognition of *E. coli* Zur [19],[20],[40],[61]. This pattern was then used as a search filter of *E. coli* (K12, strain MG1655) promoter regions to identify possible novel Zur-regulated genes using the program Fitbar [62]. The search identified four promoters, including all three of the known Zur regulated genes, and predicted a single novel Zur binding site 34 bases upstream of the periplasmic lysozyme inhibitor *pliG* gene. Alignment of the promoter sequence of the *pliG* gene to the other three known promoters showed favorable agreement with the structure-based Zur recognition profile. Previous microarray studies had demonstrated that *pliG* expression was affected by changes in Zn^2+^ concentration, however Zur regulation had not been hypothesized [34],[63]. To test whether *pliG* was in fact regulated by Zur, *pliG* transcripts were measured by reverse transcription (RT)-PCR. The presence of WTZur *in vivo*, under growth in rich media (where zinc is present in moderate to high concentrations) leads to significant repression at the *pliG* promoter (P*_pliG_*) relative to the Δ*zur* strain (Table 3). Taken together with the *in vitro* P*_pliG_* DNA-binding data (Figure 7C), these results clearly establish the *pliG* gene as part of the *E. coli* Zur regulon.

**Table 3:** Comparison of *in vitro* and *in vivo* DNA binding experiments for all four Zur promoters.

Operator	WT K_d-app_ (×10^−18^ M^2^)	Δ(ΔG°) (kcal mol^−1^)	*In Vivo* Fold Repression
*pliG*	520 ± 90	0	8.1 ± 0.6
*znuC*	8.2 ± 0.7	2.5	6.6 ± 0.9
*zinT* a	0.053 ± 0.01	5.4	984 ± 70
*L31p* a	0.025 ± 0.01	5.9	560 ± 48

*In vitro* analyses were measured by EMSA gel-shift experiments and *in vivo* experiments were performed using RT-PCR experiments monitoring the fold change in mRNA production in between both WT and Δ*zur* conditions. Δ(ΔG°) was calculated using the K_d-app_ value for each promoter and comparing the free energy to the weakest binder P*_pliG_*. This table demonstrates that both *in vivo* and *in vitro* data support the Zur hierarchy of affinity as follows: P*_L31P_* and P*_zinT_*>>P*_znuC_* and P*_pliG_*. See Data S10 for the raw data used to generate each value.

a 6 pM [DNA] was used to ensure sub-stoichiometric titrations of DNA were added to the protein-DNA samples.

### The Amplitude of *In Vivo* Zur Repression Correlates with Protein-DNA Binding Thermodynamics

We next addressed whether the degree of repression at each promoter P*_znuABC_*, P*_zinT_*, P*_L31p_*, and P*_pliG_* correlates with the affinity of Zur for the operators. Measuring the mRNA levels of the Zur regulated genes in both WT and Δ*zur* strains by RT-PCR, we find that the degree of Zur-responsive regulation varies significantly from promoter to promoter, with the most pronounced Zur-regulation seen for the ribosomal subunits and the periplasmic zinc trafficking protein. We find a clear hierarchy: Zur exhibits between 980- to 560-fold repression on *zinT* and *L31p* expression, and 7- and 8-fold repression on the *znuC* and *pliG* promoters, respectively (Table 3). The trend in these *in vivo* results correlates strongly with the relative order of *in vitro* protein-DNA affinity experiments. The binding of the two Zur dimers to form a stable DNA complex is at least 2 orders of magnitude stronger for the *zinT* and *L31p* operators than for the *znu*C and *pliG* promoters. Intriguingly, in the case of the three known Zur operators the DNA-binding occurs in a highly cooperative fashion. However, the P*_pliG_* operator seemed to interact with Zur in a different manner than the other three promoters (Figures 7C and S7). In the case of P*_pliG_* we observe a single dimer-DNA intermediate, as seen earlier in the mutant protein (R52A/D49A) titrations of P*_znuABC_*. Given that a single dimer of Zur can bind to the *pliG* operator and the precedence for single dimer Fur repression [64], it is possible that future studies will identify additional Zur operators, including those that contain only a single dimer binding site.

## Discussion

### Structure-Based Mechanism of Fur Family Repressors

To date there are 13 structurally characterized members of the Fur family of proteins across gram negative and gram positive bacteria. Each subset of the Fur family including the iron sensor proteins (Fur proteins), the nickel sensor proteins (Nur proteins), and the iron cofactor peroxide sensor proteins (PerR proteins) have been crystallized with zinc, despite the fact that they do not respond to changes in Zn^2+^ ion concentration under physiological conditions [43],[45],[47],[49]–[51],[65]. While the precise metal binding characteristics of Fur proteins remains controversial, this work provides the first structural insight into the DNA-binding characteristics for this family of proteins. The dimer of dimers structure of the Zur-*znuABC* complex explains the extended DNA footprint observed in previous experiments [26],[53]. Each Zur dimer docks on opposite sides of the DNA as predicted by previous Fur-DNA models [51],[57],[66]. Our structural characterization indicates that each dimer binds such that two successive HTH motifs contact each major groove of the DNA. Previous analysis and modeling of the winged helix motif suggested that the fourth α-helix was a “recognition helix” in the DNA-binding domain [51],[53]. Whereas our crystal structure confirms that the α4-helix (residues Pro60 to Glu72) plays a critical role in DNA-binding, residues 44–72 of the HTH motif provide the majority of the protein-DNA contacts (Figure 1B and 1C). This HTH motif includes the cooperativity linker needed for double dimerization and also Tyr45/Arg65, which make the key hydrogen bonds to the specific purine bases. In light of the (Zur_2_)_2_-DNA structure, these specific hydrogen bonds of Tyr45 and Arg65 to the purine N7 atoms can be classified as part of the Zur protein base readout mechanism of recognition [67]. Conservation of DNA backbone-contacting ligands within the Fur family, namely: Arg23, Thr25, Gln27, Arg28, and Tyr64 highlight the shape readout mechanism of backbone conformations observed for each monomer [67]. The general pattern of recognition involves several minor groove sugar and phosphate backbone contacts immediately upstream of the canonical 5′ purine and a few additional backbone interactions from the major groove at the downstream purine on the opposite strand.

A cooperative dimer-dimer repression mechanism has been described before for other metalloregulatory repressors, namely the DtxR family of repressor proteins. The prototypical member, DtxR, responds to fluctuations in Fe^2+^ concentrations in gram-positive *C. diphtheriae*, but exhibits little sequence similarity to the Fur family (<20%) [29],[68]–[71]. The similarities between the Zur and DtxR protein-DNA complexes are intriguing. Previous work highlighted the conservation of thymine bases that are critical to the recognition of the DtxR family of proteins [29],[57],[72]. Mutation of the outermost thymines of the DtxR binding site of the DNA leads to large changes in the DNA binding capability of the protein [29]. Mutation of similar thymines in the *B. subtilis* Fur protein binding sites also had a large effect on DNA binding [73]. Our structural characterization and DNA binding assays indicate that the critical contact sites are the adenines on the opposite strand, not the thymines, in the *E. coli* Zur and Fur proteins (Figure S2). In Zur, the central nucleotides play a critical role in recognition, not the outer bases as in DtxR (Figure 6C).

Native gel shift experiments reveal that the metal-saturated states of Zur and DtxR form complexes containing two dimers bound to their respective operators in a highly cooperative manner. In the case of DtxR, an intermediate containing a single dimer/DNA complex was never observed nor were any protein-protein interactions between the two dimers [29]. This leaves the molecular mode of communication between the dimers in the DtxR system as an unanswered question, including a possible role for local DNA distortion [29]. In the case of *E. coli* Zur, we have identified a pair of salt-bridges, between Asp49 and Arg52, as the key contributors to the cooperative binding of two dimers to the operator (Figure 5). This pair of acidic and basic residues is a highly conserved motif in Zur family members, but is not conserved in the Fur family overall (Table S1). Salt bridges are commonly used in protein folding and have been frequently observed as the critical junction in cooperative binding. Two noteworthy examples include the sequential oxygen binding events in hemoglobin [74],[75] and bacterial histone-like HU proteins, highlighting the prevalence of this protein-protein mode of communication [76],[77]. Whereas there are no reported protein-protein contacts between dimers or within the DNA-binding region of a Fur family member in the literature to date, two groups have identified instances where a hydrogen bond network plays a key role in Fur protein-DNA binding [43],[51]. It is likely that each subfamily of Fur proteins has some unique networks, whether hydrogen bonding or specific salt bridges to stabilize their specific protein-DNA complex.

Structure based alignments of the Zur dimers in this EcZur protein-DNA complex with several Fur family members provide strong support for a mechanism wherein metal-induced allosteric changes stimulate conversion of the wing-helix DNA-binding domain from an “open” to a “closed” conformation that is capable of binding DNA with high affinity [38],[40]–[51]. Our analysis reveals that the active DNA-binding conformation requires Zn^2+^ occupancy of both site A and site B in order to lock the key HTH motifs into the “closed” or DNA binding conformation. The effects of the mutations on the two Zn-binding sites differ. Mutational analysis of each zinc binding site demonstrates that changing even a single site A or site B amino acid dramatically reduces zinc occupancy and DNA binding affinity of EcZur (Figure 3). A mutation in site B (C88S) produces a protein capable of binding a single zinc, while a site A (C103S) mutant is unable to form stable dimers or to bind zinc under Zn^2+^-limiting conditions. These findings are in agreement with site-directed mutagenesis *in vivo* experiments using *Bs*Zur and *Sc*Zur [39],[40]. Intriguingly, in the latter two cases a third zinc binding site is observed. Previous studies of *S. coelicolor* Zur suggests that the different responses of a series of Zur regulated promoters is the result of subtle modulation of Zur-DNA binding affinities at each promoter in response to zinc availability and implicate site B and the third zinc binding sites in ScZur [40]. Our results reveal a layer of regulatory tuning based on differential affinities of zinc-saturated Zur dimers for the various promoters in this regulon. However, we cannot rule out the potential for the disordered C-terminal tail of EcZur to bind a third zinc ion thus leaving any role for this site in EcZur as an open question. This intrinsic variation, as well as the variability in the degree of cooperativity and in the number of repressor molecules binding to DNA, may contribute to the heterotropic cooperativity and differential responses in the Zur regulons of other organisms.

### All Known Promoters in the *E. coli* Zur Regulon Use a Purine-N-N-N-Pyrimidine Sequence

In the absence of Zur-DNA structures, several groups have compared Zur regulated promoters seeking consensus protein-DNA recognition patterns that could be used to define Zur regulons. This approach has been quite successful in γ and β-proteobacteria organisms, identifying as many as 23 Zur regulated genes in the case of *C. Anabaena* [25],[78],[79]. Previously the “*E. coli* Zur box” was characterized as a 23 base pair pseudo-palindromic sequence that made up the proteobacteria (gram negative) Zur regulon GAAATGTTATA-N-TATAACATTTC [25]. For the gram positive *B. subtilis*, DNA binding experiments estimated that in order for Zur to repress a promoter sequence there needed to be agreement with a similar inverted repeat 9-1-9 Zur box [80]. Using the crystallographic and biochemical data we find that the simplest motif to represent the *E. coli* Zur recognition elements involves precise purine-N-N-N-pyrimidine elements, where the monomer contacts the 5′ purine in these double strand elements. Our structure and biochemical data reveal that each Zur dimer recognizes this palindromic element with a three base spacer (RNNNYxxxRNNNY). While the number of operators is quite limited, we used this skeletal sequence to develop the sequence logo shown in Figure 7A and used it to search the *E. coli* genome and identify a novel *E. coli* Zur regulated gene, *pliG*.

This family of four operators provides a minimal basis for statistical analysis of how palindromic features of the operator sequences correlate with Zur-DNA affinity. Search programs, like SignalX, correctly identified three of the *E. coli* Zur regulated promoters. However, the positional weighted matrix ranked *znuABC* with the highest score [25]. Upon measuring the DNA-binding affinity of each of the four promoters we were surprised to find that the Zur affinity for *znuABC* is among the weakest (Table 3). The core symmetry of a Zur box is best considered as having two distinct but overlapping binding sites within the overall DNA element. Overlaying the two dimer recognition motifs using the offset seen in the crystal structure, reveals a conserved 18 bp inverted repeat RxxxYRxxR*YxxYRxxxY motif, with the * indicating the center of the palindrome (Figure 7B). When we consider the palindromic nature of the variable residues (x) and score the four known operators against the 18 bp inverted repeat we obtain the following hierarchy: L31P>ZinT≈ZnuC>>PliG. While this predicts the general pattern seen in the thermodynamic analysis, we anticipate that a more quantitative algorithm may reveal additional subtle patterns in the purine-pyrimidine repeats, which may give rise to more quantitative predictions. Furthermore, the relative number and affinity of competitive pseudosites in these AT-rich binding sites can decrease the binding free energy for the functional complex [81], and this may provide another basis for the gradation in affinities among the family of Zur regulated promoters.

### Thermodynamics of Protein-DNA Interactions Directly Correlates with Physiological Response

We find a surprisingly broad range of DNA-binding affinities (over 10,000-fold) for the known promoters in the Zur regulon (summarized in Table 3). These *in vitro* thermodynamic properties were also shown to control the functional distribution of Zn-loaded Zur over these DNA-binding sites *in vivo* (Table 3). Here the repressor-DNA affinity strongly correlates with the fold repression across the four known Zur regulated promoters in *E. coli*. These results provide strong support for the idea that the thermodynamics of an ensemble of protein-DNA interactions play a dominant role in the physiological control of gene regulation networks [81],[82]. These findings open the door to understanding how zinc availability and partial metal occupancy at a subset of the eight zinc binding sites in the repressor complex can fine tune stress responsive gene expression across a range of promoters [39],[40],[64]. Deconvolution of these factors is important for understanding how Zur and related nutrient-responsive regulons operate in pathogens that survive major shifts in local zinc concentrations induced by the host.

## Materials and Methods

### Preparation of *E. coli* Zur Proteins

WT and mutant Zur proteins were purified from *E. coli* (BL21 DE3) cells containing pET24d-based recombinant plasmid, as previously described with one minor modifications [30]. Site-directed mutagenesis was performed QuikChange mutagenesis kit and mutagenic primers (Table S2). In order to improve the purity of the Zur proteins a HiPrep Heparin fast flow column equilibrated with buffer A: 20 mM Tris-HCl (pH 8.0) and 5 mM DTT and eluted with a linear salt gradient using buffer A+500 mM NaCl. This purification step replaced of the ammonium sulfate purification step described earlier in [30]. The molecular weights of purified WT and mutant Zur proteins were confirmed by electrospray ionization mass spectrometry (ESI-MS).

### Protein-DNA Complex Crystallization and Data Collection

Zur protein and the designed DNA were mixed together to a final concentration of [Zur] = 117 µM, [DNA] = 108 µM, with [DTT] = 5 mM and [ZnSO_4_] = 150 µM. The optimal DNA for crystallography included 31 nucleotides known to be protected in footprinting assays [26] with a 3′ overhang of two bases to help stabilize the crystal packing, referred to as 31mer2bpOH. Over 40 sequences of DNA were tested using hanging drop vapor diffusion and 31mer2bpOH led to the most reproducible high quality diffraction data. Crystals were grown by the hanging drop vapor diffusion method, mixing 1 µl of protein/DNA mixture with 1 µl of crystallization buffer (1.5 M ammonium sulfate, 0.1 M citrate [pH 5.5]). Crystal trays were set up and incubated at 4°C. Crystals grew to full size in 2 weeks. The crystals were cryoprotected with mother liquor supplemented with 15% ethylene glycol and flash-cooled in liquid nitrogen. Data were collected at 100 K at the Advanced Photon Source (APS) Life Sciences Collaborative Access Team (LS-CAT) and diffracted to 2.5 Å, crystallographic statistics are summarized in Table 1.

### Structure Determination

X-ray diffraction data were processed using the programs XDS and SCALA to 2.5 Å resolution with a C2 space group and a unit cell *a* = 193.4, *b* = 80.5, *c* = 98.8, and α = **γ** = 90.0 and β = 120.2. The structure was solved by using multiwavelength anomalous dispersion from data collected at the zinc absorption edge (9.7 keV) and a higher energy dataset (12.7 keV). The positions of eight zinc atoms were found and refined using SHARP/autoSHARP [83],[84]. The derived experimental phases were improved by solvent flattening and revealed clear density for DNA and protein regions. The model was built with Coot and refined using REFMAC5 [85],[86] and Phenix [87]. The final model validated by MOLPROBITY has good geometry and most of the residues are in favorable regions in the Ramachandran plot [88]. All images from the Zur crystal structure were rendered using PYMOL [89].

The dimer of dimers nature of protein-DNA interaction made it impossible to assign the proper directionality of the DNA electron density *de novo*. In order to ascertain whether the Zur protein recognized a single DNA direction over the other, the Zur-DNA protein complex was crystallized with brominated DNA. The coding strand of 31mer2bpOH brominated at positions 8 and 22 of the DNA was ordered commercially in HPLC purified grade from IDT. The brominated DNA pellet was resuspended with MiliQ H_2_O (Millipore) annealed with non-brominated HPLC purified 31mer2bpOH non-coding strand. The protein-DNA complex was prepared by mixing the following components to a final concentrations of [Zur] = 120 µM, [Br-31mer2bp OH] = 120 µM, [ZnSO_4_] = 150 µM, and DTT = 5 mM. Crystals were grown in the same reservoir solution as non-brominated DNA (1.5 M ammonium sulfate, 0.1 M citrate pH 5.5) and 15% ethylene glycol was used a cryoprotectant. Brominated crystal diffraction data were collected at 13.5 keV (Bromine K edge) and diffracted to 3.5 Å.

### Preparation of Fluorescently Labeled DNA

Fluorescently labeled DNA was isolated using PCR amplification of 51 bases from the ZnuC footprinting plasmid pUC19-Znufoot [26]. Two primers Cy5-Znu-forward and Cy5-Znu-reverse (ordered from IDT [Integrated DNA Technologies]) was used to amplify only a portion of this plasmid. The annotation “/5Cy5/” corresponds to the single Cy5 fluorescent probe covalently attached to the 5′ end of the DNA. The PCR reaction was carried out in 25 µl reaction volume with 20–50 ng of plasmid, 1× Thermopol buffer (New England Biolabs [NEB]), 10 µM of each primer, and 200 µM of each dNTP and 1 unit of DeepVent DNA polymerase (NEB). After 15 cycles of amplification the reaction was run on a 2% w/v agarose gel at 130 volts for 1.5 h. The DNA bands were visualized using UV excitation of the ethidium bromide mixed into the gel and the 51 bp fluorescent product was excised from the agarose gel using a razor blade. The DNA was solubilized using a QIAEX II Gel Extraction kit (Qiagen). Once the DNA was isolated, the concentration was estimated by running a small sample (5 µl) of the newly isolated DNA product, which was run on a new 2% agarose gel along with 5 dilutions of the 100 bp DNA ladder (NEB). The 100 bp ladder contained established DNA concentrations and the intensity of these bands was compared to that of the newly isolated DNA product. Typical PCR reactions lead to approximately 40 µl of ∼0.2 µM fluorescently labeled DNA.

In order to test the effect of mutating the protein contact sites on the *znuABC* promoter, site directed mutagenesis (Stratagene) was performed on the pUC19-Znufoot plasmid using mutagenic primers (Table S2). This plasmid had previously been used to generate the 340 bp sequence for Zur footprinting experiments [26]. Each of the mutations was confirmed by DNA sequencing using universal M13 primers. Once the mutation was confirmed fluorescently labeled mutant *znuABC* promoter sequences were isolated in the same manner as the WT sequence described above.

### Electrophoretic Mobility Shift Assays

All electrophoretic mobility shift assay (EMSA) experiments were run on a 10% polyacrylamide gel containing 0.1 M Tris (pH 8.3) 0.1 M boric acid, and 50 µM ZnSO_4_. For EMSA experiments the DNase I footprinting buffer used by Outten and colleagues [26] was modified in order to weaken the interaction between protein and DNA. Weakening the affinity between protein and DNA allowed us to use DNA concentrations high enough to obtain a reasonable signal, while ensuring that [DNA]<<K_d-app_. Both protein and DNA were diluted in the EMSA binding buffer, which contained 10 mM Tris (pH 8.0), 10 mM NaCl, 100 mM L-potassium glutamic acid, 1.5 mM MgCl_2_, 1 mM CaCl_2_, 0.1 µg/ml bovine serum albumin (BSA), 1 mM dithiothreitol (DTT), 2 µg/ml sonicated salmon sperm DNA, 50 µM ZnSO_4_, and 167 mM KCl. DNA fragments (≤45 pM) were mixed together with varying concentrations of Zur protein at 22°C for 30 min, and 85 µl of the 90 µl total volume was loaded directly onto a running gel (constant 145 volts) for 3 h.

Using a Typhoon 9410 Variable Mode imager (GE) each EMSA gel was excited at a wavelength of 633 nm and the intensity of the fluorescent emission at 690 nm was used to identify the presence of the labeled DNA. Apparent affinities were measured by comparing the ratio of bound (shifted species) to that of the free fluorescently labeled DNA within the gel. The ratios of free versus bound DNA were determined by using the imaging software ImageJ. Using the ImageJ profile of each lane in the EMSA gel, the fraction of shifted DNA species was plotted against the concentration of the protein sample. In order to calculate the apparent dissociation constants from the gel-shift data, the models of equilibrium binding in Equations 2a and 2b were fit to the data from two independent experiments using a non-linear least squares regression in MATLAB [90]. The fmincon optimization engine in MATLAB was used with the Interior Point minimization algorithm with default parameters. The standard error of the best fit parameters were calculated using the asymptotic standard error of the best fit determined from GraphPad Prism using the models in Equations 2a and 2b mentioned in the Results section.

### RT-PCR assays

RT-PCR was conducted to measure mRNA level changes in *E. coli* MG1655 WT and Δ*zur* cells. Cells were grown in LB medium into mid-log phase, and collected by centrifugation. Total RNA was purified by Qiagen RNA miRNeasy kit. RNA concentrations and quality were determined by NanoDrop Lite spectrophotometer (Thermal Scientific) and by 2100 Bioanalyzer (Agilent Technologies). The primers used for RT-PCR were ordered from Integrated DNA Technologies (IDT). The complete list of the primers and their sequences is shown below: zinT18_up (5′- CAAACTGGCTGTTGCTTTAGG-3′), zinT104_dn (5′-TCTGTTAAGGGTTTGCCGTG-3′), pliG7_up(5′-ATCAAGAGCATCAGGAAGGC-3′), pliG85_dn(5′-CATTGACATTCTTACCCGCAG-3′), znuC220_up (5′-CAGAAGCTGTATCTCGACACC-3′), znuC297_dn (5′-TTCTTTATGTGTACCAGGGCG-3′), ykgM90_up (5′-AACAGACCGTGAGATTGAGC-3′), ykgM204_dn (5′-TCCTTCTGATGCCACTGTTC-3′). The efficiency of all primer pairs were tested to be within 95% to 105%. The reagent for RT-PCR was the iTaq Universal SYBR Green One-Step kit with SYBR green (Bio-Rad), and the instrument used was the iQ5 (Bio-Rad). Each reaction contained 10 ng of total RNA and 300 nM of each primer.

### Data Deposition

Data pertaining to the experiments in this article have been deposited in the Dryad repository at http://dx.doi.org/10.5061/dryad.vn6dv [91].

### Accession Numbers

Protein Data Bank (http://www.rcsb.org/pdb): coordinates and structure factors for the Zur-DNA-Zn complex have been deposited in the Protein Data Bank with accession codes: 4MTD (Orientation 1) and 4MTE (both orientations).

## Supporting Information

Figure S1 **DNA-Binding of A-site (C103S) and B-site (C88S) mutant under excess Zinc.** DNA binding activity of mutant Zur proteins analyzed by EMSA gel shifts of the *znuABC* operator in the presence of 50 µM ZnSO_4_. Using these qualitative gel shift experiments it is apparent that even in the presence of excess zinc a single site-directed mutation in site A or site B retains a dramatic weakening of the Zur DNA-binding affinity (WTZur saturation of binding ca. 10 nM Dimer).
(TIF)

Figure S2 **Key amino acids interacting with P** ***_znuABC_*** **DNA bases.** (A) Arg65 and (B) Tyr45 side chains interacting with the DNA bases. The figures highlight the specific hydrogen bonding that occurs between both the arginine and tyrosine side chains and the respective purines N7 nitrogen atoms that they interact with.
(TIF)

Figure S3 **Determination of the stoichiometry of Zur-DNA complexes by native PAGE.** Logarithms of the relative mobilities of Zur-DNA and standard proteins (compared to the mobility of bromophenol blue) against percentage of acrylamide concentration using a previously established protocol [57]. The samples tested were Zur_2_- P*_pliG_* (▾, purple), Zur_2_(R52A)-P*_znuABC_* (•, grey), (Zur_2_)_2_- P*_pliG_* (▴, pink), and (Zur_2_)_2_- P*_znuABC_* (♦, light green). The four protein standards that were used were chicken egg white lysozyme (○, red), bovine serum albumin monomer (□, purple), bovine serum albumin dimer (▵, dark green), and β-amylase (▽, orange). Determination of the apparent molecular weight were calculated using a (A) plot of the negative slopes of mobility against the known molecular weight of the four standards. (B) Using least squares regression for the predicted molecular weights, the Zur_2_-DNA predicted weight was shown to be 70 kDa and 110 kDa for the (Zur_2_)_2_-DNA complex. These values were within experimental error of the theoretical molecular weights of 71 and 110 kDa for the 1∶1 and 2∶1 protein: DNA complexes, respectively. See Data S5 for the raw data used to generate each plot.
(TIF)

Figure S4 **Hill plot measures the central portion of WT Zur-P** ***_znuABC_*** **gel shifts.** Measure of the cooperativity from the EMSA binding between WT Zur (0.75–10 nM) and P*_znuABC_* operator. Using the slope from the line of best fit the Hill coefficient (α_H_) can be estimated to be ∼2.1. Hill plots for all four WT operators generated α_H_>1, corresponding to a cooperative binding interaction. See Data S6 for the raw data used to generate this plot.
(TIF)

Figure S5 **Effect of Zur(R52A) mutation on DNA binding.** (A) Native gel shifts demonstrate the isolation of a single dimer-DNA intermediate in mutant protein (R52A) unseen in the WT Zur gel shifts. Shown here is a representative gel-shift for Zur(R52A)_2_ titration of P*_znuABC_*. (B) Two-site binding isotherms modeled for the equilibrium for Zur(R52A)_2_ binding corresponding to K_d1_ = 2.6 nM (orange) and K_d2_ = 220 nM (blue). Taken together gel shifts of the cooperativity linker mutants demonstrate the binding of the first dimer significantly weakens the binding of the second dimer. See Data S7 for the raw data used to generate this plot.
(TIF)

Figure S6 ***E. coli*** **Zur binds to wide major and narrow minor groove widths.** (A) Phosphate backbone trace of the *znuABC* DNA in the Zur_2_-DNA crystal structure. This trace highlights the wide major grooves and narrow minor grooves in the center of the DNA molecule. (B) Comparison of the major and minor groove in the *znuABC* DNA. All groove width calculations were performed using Curves+ [58]. The major groove steps are shown in blue and are frequently larger than the major groove of ideal canonical B-form DNA. Variations in the major and minor groove width are categorized as key recognition elements to DNA binding proteins [67]. The largest major groove is located at the center of the DNA (base 15 and 16). The minor groove is narrowed when compared to ideal DNA and the smallest minor groove occurs at the central bases of DNA. See Data S8 for the raw data used to generate this plot.
(TIF)

Figure S7 **Affinity determination of WT Zur titrations of P** ***_pliG_*** **by EMSA.** Native gel shifts demonstrate the isolation of a single dimer-DNA intermediate in unseen in other WT Zur gel shifts. Fits for the dual macroscopic binding constants for the single dimer (orange) and double dimer (light blue) species were obtained using Equation 2a. The binding constants are estimated as K_d1_ = 28 nM and K_d2_ = 19 nM. This observation of the single-dimer intermediate formation using WT Zur titrations highlights the unique nature of Zur-*pliG* interactions. See Data S9 for the raw data used to generate this plot.
(TIF)

Table S1 **Sequence based conservation of DNA-binding and salt-bridge amino acids.** Alignment of *E. coli* Zur Tyr45 and Arg65, which make the key hydrogen bond donations to the purines of the DNA. Also highlighted are the alignments of the dimer-dimer salt-bridge amino acids (Asp49/Arg52) in gram-negative and positive Zur and Fur protein sequences. Each protein was individually aligned using ClustalW [95] with the *E. coli* Zur sequence in order to monitor the conservation of both sets of amino acids. In general, both DNA-binding and salt-bridge linkers are conserved amongst gram-negative Zur proteins. While Arg65 is highly conserved across many members of the Fur family of proteins, even Mur and Nur (alignment not shown), there is little to no conservation of the cooperativity linkers found in their Zur counterparts.
(DOCX)

Table S2 **Primers used for plasmid construction and gel-shift DNA.**
(DOCX)

Data S1 **Raw data from** **Figure 3** **.** File includes raw data used in inductively coupled plasma mass spectrometry (ICP-MS) and *in vivo* complementation experiments.
(XLSX)

Data S2 **Raw data from** **Figure 4** **.** File includes raw data used to determine the apparent K_d_ calculations of the WTZur gel-shift experiments with P_znuABC_ DNA.
(XLSX)

Data S3 **Raw data from** **Figure 5** **.** File includes raw data used to determine the apparent K_d_ calculations for mutant Zur(D49A) gel-shift experiments with P_znuABC_ DNA.
(XLSX)

Data S4 **Raw data from** **Figure 6** **.** File includes raw data used to determine the apparent K_d_ calculations of the WTZur gel-shift experiments with mutated P_znuABC_ DNA, along with additional Hill plots used to monitor the cooperativity of binding.
(XLSX)

Data S5 **Raw data from Figure S3.** Summary of raw data from Figure S3 Native gel shift experiments using varying percentages of acrylamide.
(XLSX)

Data S6 **Raw data from Figure S4.** Raw data from Hill plot used in Figure S4.
(XLSX)

Data S7 **Raw data from Figure S5.** File includes raw data used to determine the apparent K_d_ calculations for mutant Zur(R52A) gel-shift experiments with P_znuABC_ DNA.
(XLSX)

Data S8 **Raw data from Figure S6.** Raw data from major and minor groove width Curves+ analysis in Figure S6.
(XLSX)

Data S9 **Raw data from Figure S7.** File includes raw data used to determine the apparent K_d_ calculations of the WTZur gel-shift experiments with P_pliG_ DNA.
(XLSX)

Data S10 **Raw data from** **Table 3** **.** File includes raw data used to determine the apparent K_d_ calculations of the WTZur gel-shift experiments with P_pliG_, P_znuABC_, P_zinT_, and P_L31p_ DNA. Data also includes the raw data from the *in vivo* repression data of the four *E. coli* Zur regulated operators.
(XLSX)

Scheme S1 **Derivation of the stepwise microscopic equilibrium expressions.** Mass-balance calculation for the stepwise binding events for (Zur_2_)_2_-DNA binding.
(DOCX)

## Abbreviations

31mer2bpOH: DNA with a core of 31 bps and a 2 bp overhang
DtxR: diphtheria toxin repressor
EcZur: *E. coli* Zur
EMSA: electron mobility shift assay
Fur: ferric uptake regulator
HTH: helix-turn-helix
K_d-app_: macroscopic dissociation constant
PerR: peroxide repressor
pliG: periplasmic lysozyme inhibitor
RT-PCR: reverse transcription PCR
WT: wild-type
WTZur: wild-type Zur
Zur: zinc uptake regulator
α_H_: Hill coefficient
