# Supplementary figures and images for: Structural and Mechanistic Basis of Zinc Regulation Across the E. coli Zur Regulon
Source: PLoS Biol. 2014 Nov 4;12(11):e1001987. doi: 10.1371/journal.pbio.1001987 (PMC4219657; doi:10.1371/journal.pbio.1001987)

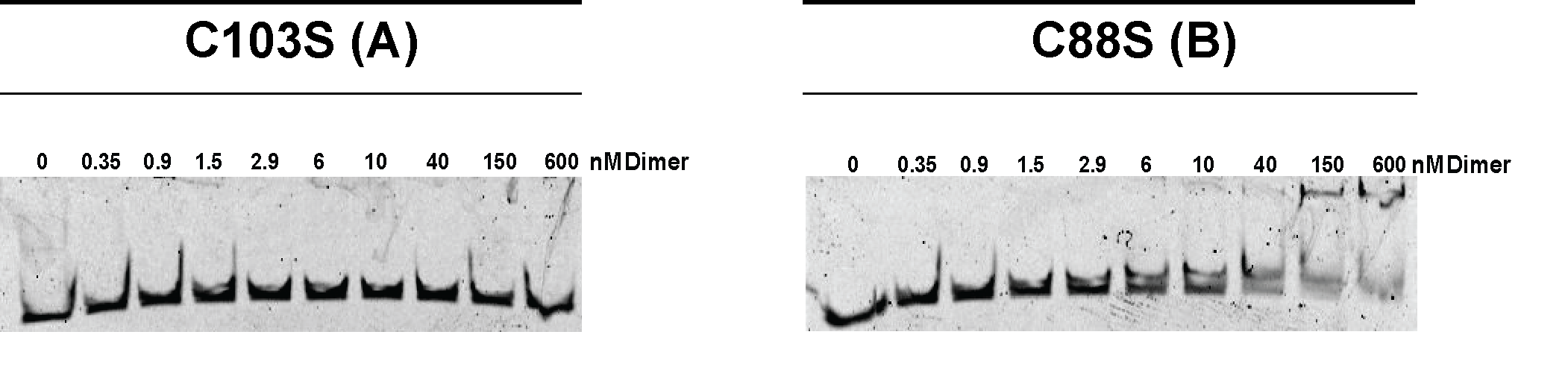

Supplement: Figure S1 — DNA-Binding of A-site (C103S) and B-site (C88S) mutant under excess Zinc. DNA binding activity of mutant Zur proteins analyzed by EMSA gel shifts of the znuABC operator in the presence of 50 µM ZnSO4. Using these qualitative gel shift experiments it is apparent that even in the presence of excess zinc a single site-directed mutation in site A or site B retains a dramatic weakening of the Zur DNA-binding affinity (WTZur saturation of binding ca. 10 nM Dimer). (TIF) [file pbio.1001987.s001.tif]

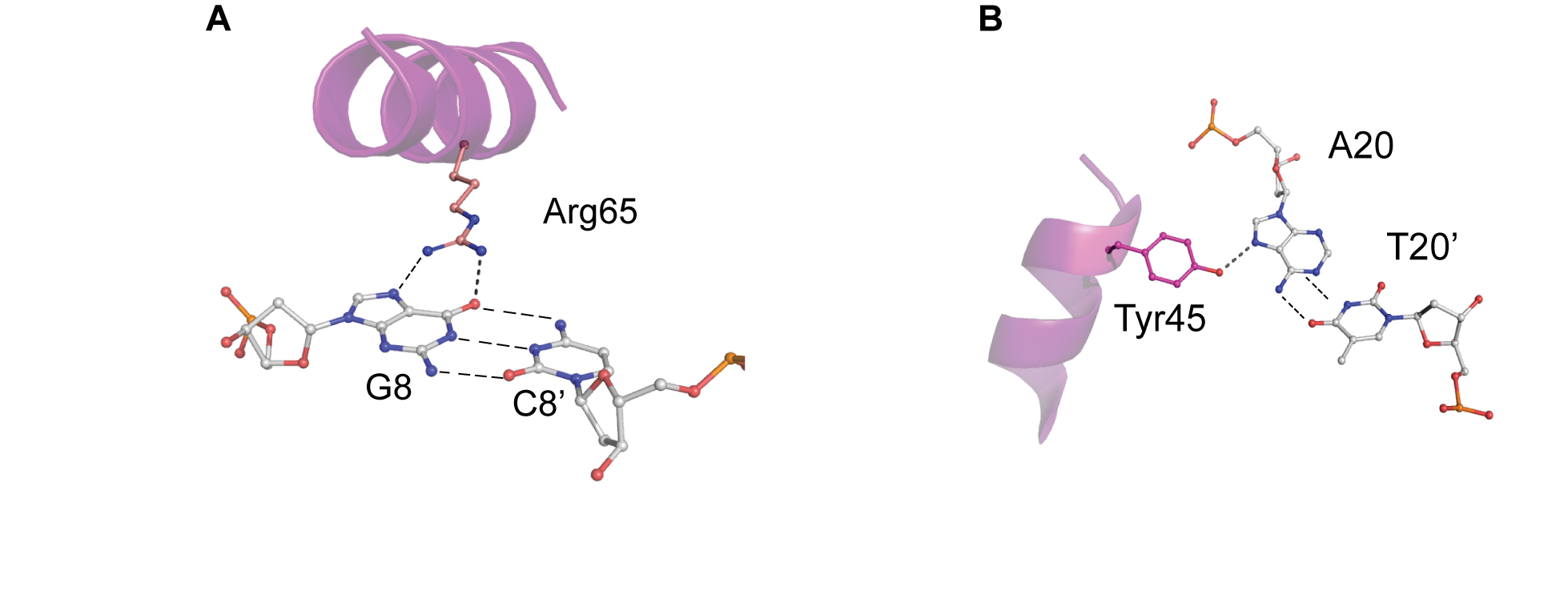

Supplement: Figure S2 — Key amino acids interacting with P znuABC DNA bases. (A) Arg65 and (B) Tyr45 side chains interacting with the DNA bases. The figures highlight the specific hydrogen bonding that occurs between both the arginine and tyrosine side chains and the respective purines N7 nitrogen atoms that they interact with. (TIF) [file pbio.1001987.s002.tif]

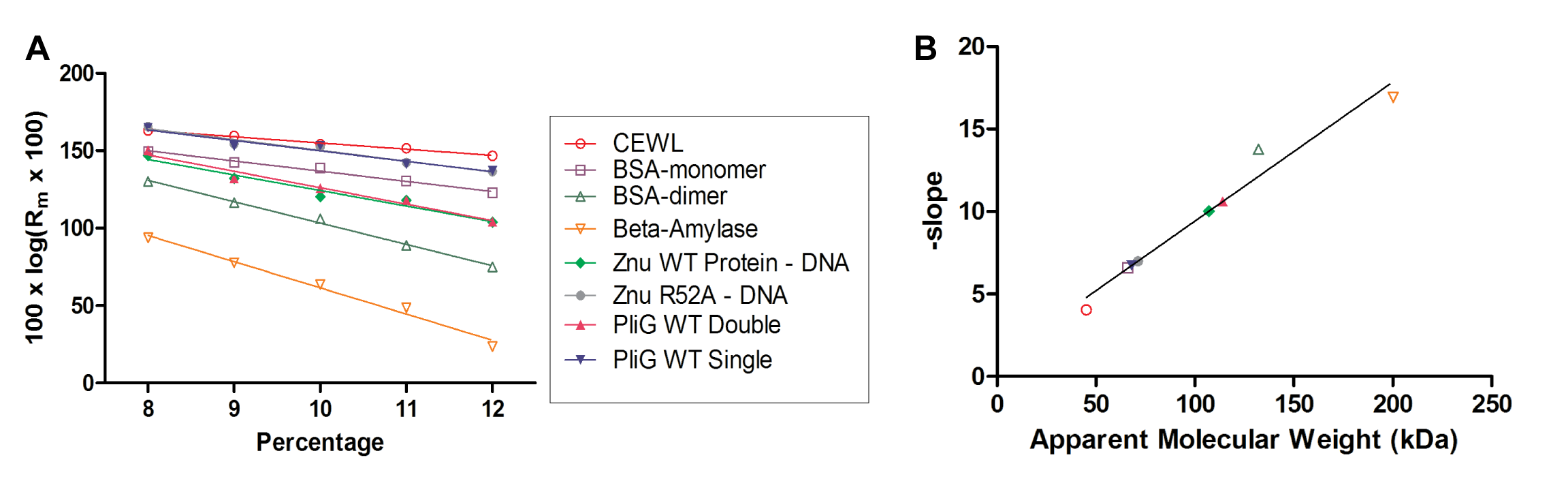

Supplement: Figure S3 — Determination of the stoichiometry of Zur-DNA complexes by native PAGE. Logarithms of the relative mobilities of Zur-DNA and standard proteins (compared to the mobility of bromophenol blue) against percentage of acrylamide concentration using a previously established protocol [57]. The samples tested were Zur2- PpliG (▾, purple), Zur2(R52A)-PznuABC (•, grey), (Zur2)2- PpliG (▴, pink), and (Zur2)2- PznuABC (♦, light green). The four protein standards that were used were chicken egg white lysozyme (○, red), bovine serum albumin monomer (□, purple), bovine serum albumin dimer (▵, dark green), and β-amylase (▽, orange). Determination of the apparent molecular weight were calculated using a (A) plot of the negative slopes of mobility against the known molecular weight of the four standards. (B) Using least squares regression for the predicted molecular weights, the Zur2-DNA predicted weight was shown to be 70 kDa and 110 kDa for the (Zur2)2-DNA complex. These values were within experimental error of the theoretical molecular weights of 71 and 110 kDa for the 1∶1 and 2∶1 protein: DNA complexes, respectively. See Data S5 for the raw data used to generate each plot. (TIF) [file pbio.1001987.s003.tif]

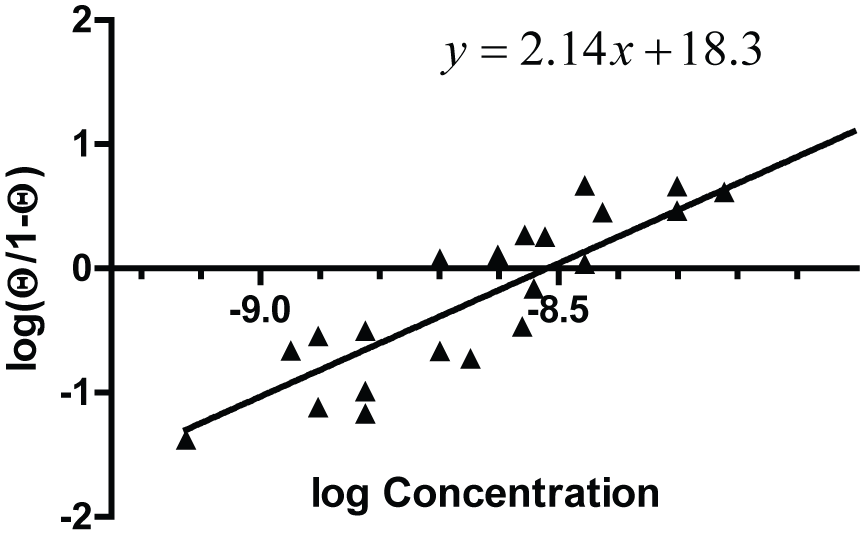

Supplement: Figure S4 — Hill plot measures the central portion of WT Zur-P znuABC gel shifts. Measure of the cooperativity from the EMSA binding between WT Zur (0.75–10 nM) and PznuABC operator. Using the slope from the line of best fit the Hill coefficient (αH) can be estimated to be ∼2.1. Hill plots for all four WT operators generated αH>1, corresponding to a cooperative binding interaction. See Data S6 for the raw data used to generate this plot. (TIF) [file pbio.1001987.s004.tif]

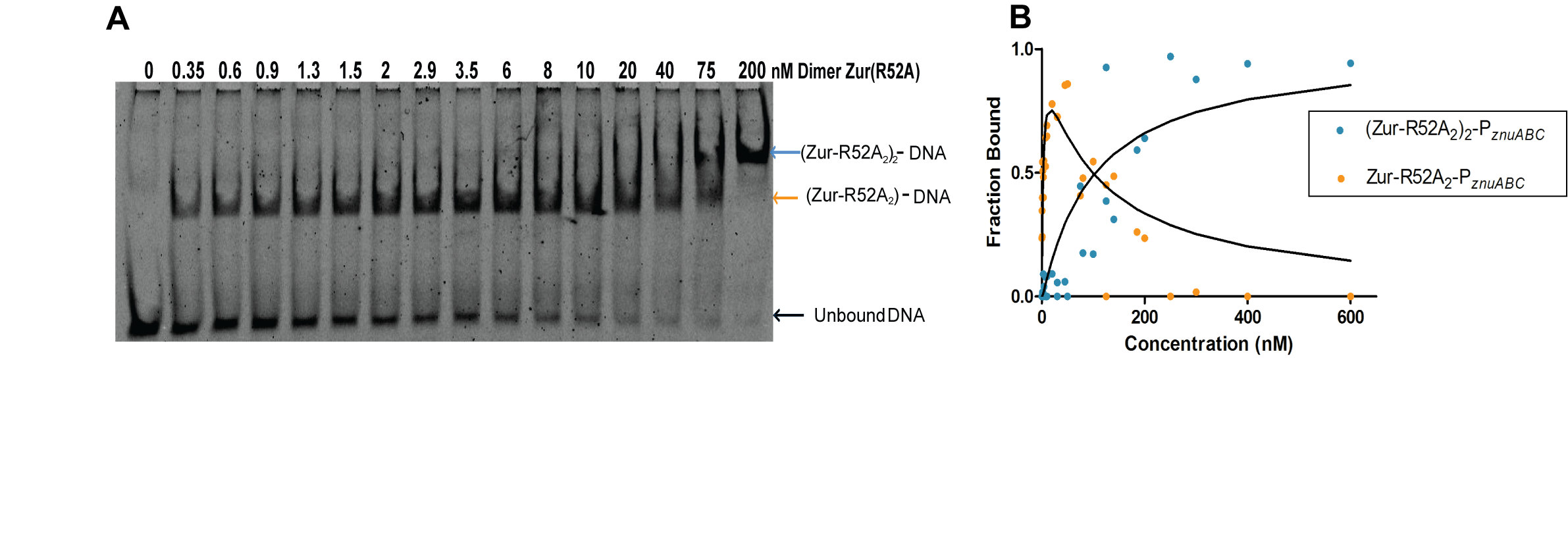

Supplement: Figure S5 — Effect of Zur(R52A) mutation on DNA binding. (A) Native gel shifts demonstrate the isolation of a single dimer-DNA intermediate in mutant protein (R52A) unseen in the WT Zur gel shifts. Shown here is a representative gel-shift for Zur(R52A)2 titration of PznuABC. (B) Two-site binding isotherms modeled for the equilibrium for Zur(R52A)2 binding corresponding to Kd1 = 2.6 nM (orange) and Kd2 = 220 nM (blue). Taken together gel shifts of the cooperativity linker mutants demonstrate the binding of the first dimer significantly weakens the binding of the second dimer. See Data S7 for the raw data used to generate this plot. (TIF) [file pbio.1001987.s005.tif]

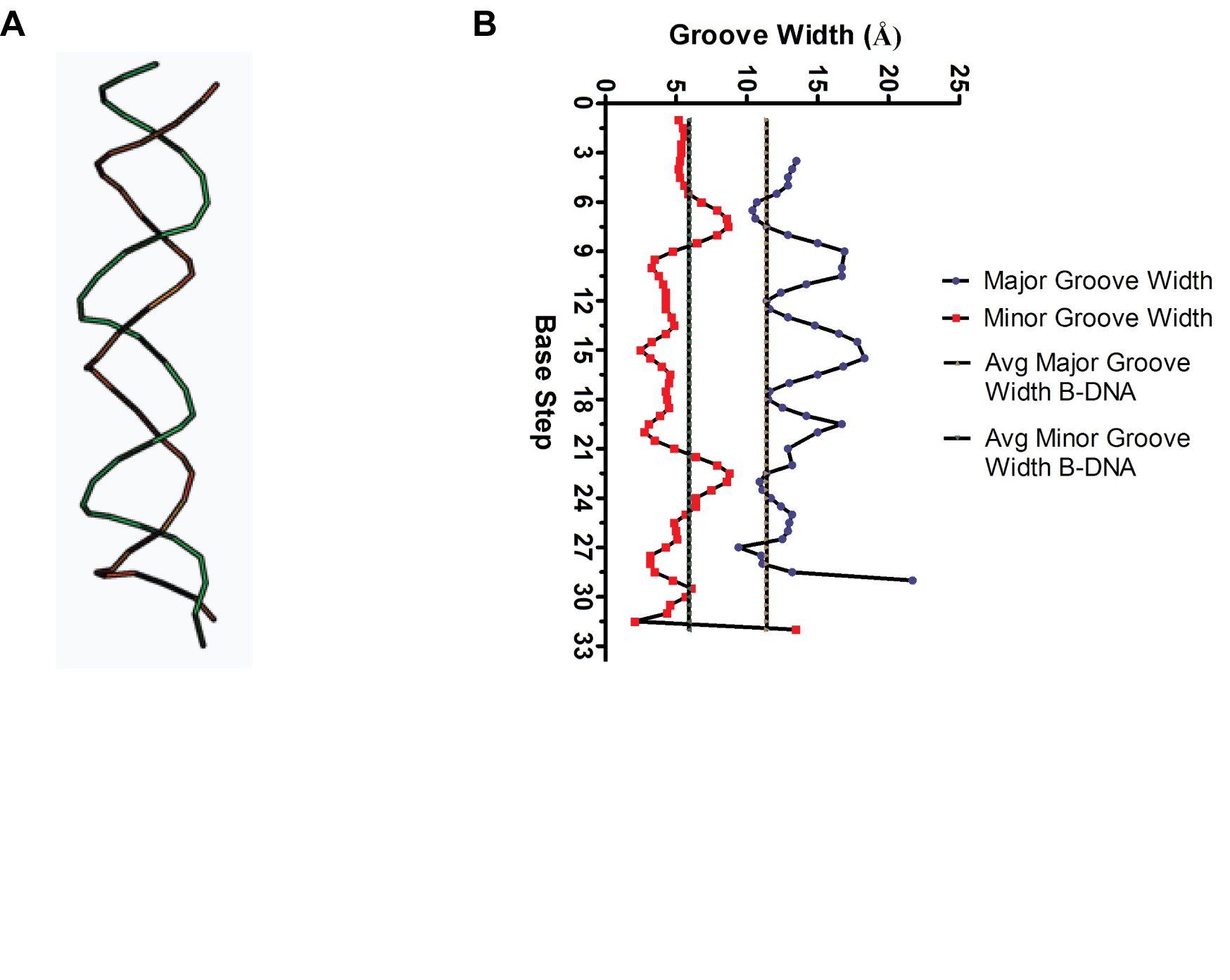

Supplement: Figure S6 — E. coli Zur binds to wide major and narrow minor groove widths. (A) Phosphate backbone trace of the znuABC DNA in the Zur2-DNA crystal structure. This trace highlights the wide major grooves and narrow minor grooves in the center of the DNA molecule. (B) Comparison of the major and minor groove in the znuABC DNA. All groove width calculations were performed using Curves+ [58]. The major groove steps are shown in blue and are frequently larger than the major groove of ideal canonical B-form DNA. Variations in the major and minor groove width are categorized as key recognition elements to DNA binding proteins [67]. The largest major groove is located at the center of the DNA (base 15 and 16). The minor groove is narrowed when compared to ideal DNA and the smallest minor groove occurs at the central bases of DNA. See Data S8 for the raw data used to generate this plot. (TIF) [file pbio.1001987.s006.tif]

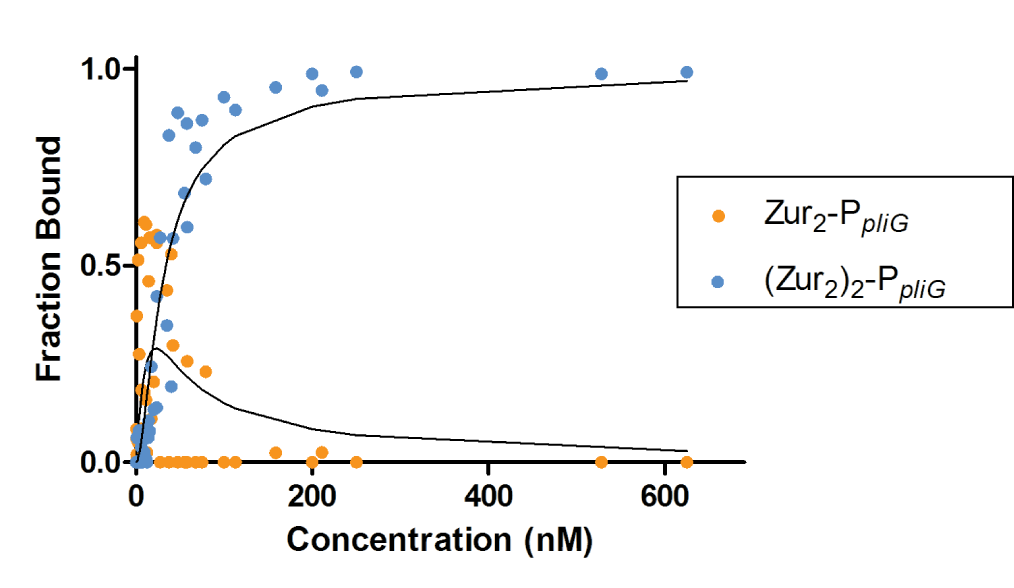

Supplement: Figure S7 — Affinity determination of WT Zur titrations of P pliG by EMSA. Native gel shifts demonstrate the isolation of a single dimer-DNA intermediate in unseen in other WT Zur gel shifts. Fits for the dual macroscopic binding constants for the single dimer (orange) and double dimer (light blue) species were obtained using Equation 2a. The binding constants are estimated as Kd1 = 28 nM and Kd2 = 19 nM. This observation of the single-dimer intermediate formation using WT Zur titrations highlights the unique nature of Zur-pliG interactions. See Data S9 for the raw data used to generate this plot. (TIF) [file pbio.1001987.s007.tif]
