# Supplementary material for: Structural and Mechanistic Basis of Zinc Regulation Across the E. coli Zur Regulon
Source: PLoS Biol. 2014 Nov 4;12(11):e1001987. doi: 10.1371/journal.pbio.1001987 (PMC4219657; doi:10.1371/journal.pbio.1001987)
Supplement: Table S1 — Sequence based conservation of DNA-binding and salt-bridge amino acids. Alignment of E. coli Zur Tyr45 and Arg65, which make the key hydrogen bond donations to the purines of the DNA. Also highlighted are the alignments of the dimer-dimer salt-bridge amino acids (Asp49/Arg52) in gram-negative and positive Zur and Fur protein sequences. Each protein was individually aligned using ClustalW [95] with the E. coli Zur sequence in order to monitor the conservation of both sets of amino acids. In general, both DNA-binding and salt-bridge linkers are conserved amongst gram-negative Zur proteins. While Arg65 is highly conserved across many members of the Fur family of proteins, even Mur and Nur (alignment not shown), there is little to no conservation of the cooperativity linkers found in their Zur counterparts. (DOCX) [file pbio.1001987.s008.docx]

| **Protein Analyzed** | **Alignment of DNA-binding residues Tyr45/ Arg65** | **Alignment of Salt Bridges Asp49/Arg52** | **Sequence Identity to**  ***E. coli* Zur (%)** |
| --- | --- | --- | --- |
| **Gram Negative Zur** |  |  |  |
| *S. typhi* | Tyr / Arg | Asp / Arg | 93 |
| *K. pneumoniae* | Tyr / Arg | Asp / Arg | 87 |
| *Y. pestis* | Tyr / Arg | Asp / Arg | 69 |
| *V. cholerae* | Tyr / Arg | Glu / Lys | 49 |
| *A. tumefaciens* | Tyr / Arg | Asp / Arg | 37 |
| *B. melitensis* | Tyr / Arg | Asp / Arg | 36 |
| *S. meliloti* | Tyr / Arg | Asp / Arg | 39 |
| *M. loti** | Tyr / Arg | Asp / Arg | 43 |
| **Gram Negative Fur** |  |  |  |
| *E. Coli* | Glu / Arg | Lys / Ile | 28 |
| *S. typhi* | Glu / Arg | Lys / Ile | 27 |
| *K. pneumoniae* | Glu / Arg | Lys / Ile | 27 |
| *Y. pestis* | Glu / Arg | Lys / Ile | 27 |
| *V. cholerae* | Glu / Arg | Lys / Ile | 27 |
| *A. tumefaciens* | Glu / Arg | Arg / Ser | 24 |
| *S. meliloti* | Ser / Arg | Arg / Lys | 27 |
|  |  |  |  |
| **Gram Positive Zur** |  |  |  |
| *S. Coelicolor* | Gln / Arg | Asp / Lys | 29 |
| *M. tuberculosis* | Gln / Arg | Asp / Arg | 30 |
| *B. subtilis* | Lys / Arg | Ser / Asn | 24 |
| *B. halodurans* | Asp / Asn | Lys / Glu | 28 |
| *B. anthracis* | Lys / Arg | Glu / Lys | 23 |
| *L. monocytogenes* | Lys / Arg | Glu / Lys | 23 |
| *S. aureus* | Lys / Arg | Gln / Asp | 23 |
| *E. faecalis* | Asp / Asn | Arg / Glu | 27 |
| **Gram Positive Fur** |  |  |  |
| *S. Coelicolor* | Glu / Asp | Ser / Arg | 25 |
| *M. tuberculosis* | Glu / Asp | Gly / Arg | 24 |
| *B. subtilis* | Glu / Arg | Leu / Arg | 29 |
| *B. halodurans* | Asp / Asn | Lys / Glu | 28 |
| *L. monocytogenes* | Glu / Arg | Leu / Lys | 27 |
| *S. aureus* | Glu / Arg | Leu / Lys | 25 |
| *E. faecalis* | Glu / Arg | Phe / Lys | 24 |
|  |  |  |  |

*Our alignment suggests that *M. loti* Fur family transcriptional regulator should be characterized as a Zur protein, not as a Fur protein.
