# Supplementary material for: Structural and Mechanistic Basis of Zinc Regulation Across the E. coli Zur Regulon
Source: PLoS Biol. 2014 Nov 4;12(11):e1001987. doi: 10.1371/journal.pbio.1001987 (PMC4219657; doi:10.1371/journal.pbio.1001987)
Supplement: Table S2 — Primers used for plasmid construction and gel-shift DNA. (DOCX) [file pbio.1001987.s009.docx]

| **Primer** | **Sequence (5′ to 3′)^a^** |  |
| --- | --- | --- |
| **Crystallography** | |  |
| 31mer2bpOH-f | AGAAGTGTGATATTATAACATTTCATGACTATG |  |
| 31mer2bpOH-r | TAGTCATGAAATGTTATAATATCACACTTCTCA |  |
| Br-31mer2bpOH-f ^b^ | AGAAGTG/dU-Br/GATATTATAACAT/dU-Br/TCATGACTATG |  |
| Br-31mer2bpOH-r | TAGTCATGAAATGTTATAATATCACACTTCTCA |  |
|  |  |  |
| **Mutagenesis of Pznu plasmid** | | Template:  pUC19-ZnuABC |
| znu11TA-f | GTAATGAATATGAGAAGTGTGAAATTATAACATTTCATGACTAC |  |
| znu11TA-r | GTAGTCATGAAATGTTATAATTTCACACTTCTCATATTCATTAC |  |
| znu12AT-f | GAATATGAGAAGTGTGATTTTATAACATTTCATGACTACTG |  |
| znu12AT-r | CAGTAGTCATGAAATGTTATAAAATCACACTTCTCATATTC |  |
| znu15AT-f | GAATATGAGAAGTGTGATATTTTAACATTTCATGACTACTGC |  |
| znu15AT-r | GCAGTAGTCATGAAATGTTAAAATATCACACTTCTCATATTC |  |
| znu16TA-f | GAATATGAGAAGTGTGATATTAAAACATTTCATGACTACTGCAAG |  |
| znu16TA-r | CTTGCAGTAGTCATGAAATGTTTTAATATCACACTTCTCATATTC |  |
| znu19CG-f | GAGAAGTGTGATATTATAAGATTTCATGACTACTGCAAGAC |  |
| znu19CG-r | GTCTTGCAGTAGTCATGAAATCTTATAATATCACACTTCTC |  |
| znu20AT-f | GAGAAGTGTGATATTATAACTTTTCATGACTACTGCAAGAC |  |
| znu20AT-f | GTCTTGCAGTAGTCATGAAAAGTTATAATATCACACTTCTC |  |
|  |  |  |
|  |  |  |
| **Mutagenesis of Zur overexpression vector** | | pET24d-Zur |
| R52A-f | GATTTACTGGCCGAAGCTGAACCG |  |
| R52A-r | CGGTTCAGCTTCGGCCAGTAAATC |  |
| D49A-f | ATGATCTGCTTGCTTTACTGCGCG |  |
| D49A-r | CGCGCAGTAAAGCAAGCAGATCAT |  |
| C88S-f | CAGTTATGTGCTCTCTCATCTGTTCGATC |  |
| C88S-r | GATCGAACAGATGAGAGAGCACATAACTG |  |
| C103S-f | CATGTTTATTAGCGATCGCTGCGGC |  |
| C103S-r | GATCGCTAATAAACATGGCTGACG |  |
| **Fluorescent PCR for Mutant Gel Shifts** | | pUC19-ZnuABC |
| Cy5-Znu-forward | /5Cy5/CCGAATATGAGAAGTGTG^c^ |  |
| Cy5-Znu-reverse | CCGTCTTGCAGTAGTCA |  |
|  |  |  |
| **Fluorescent Probes for Wild-type Gel Shifts** | |  |
| Cy5-znuABC-f | /5Cy5/CCGAATATGAGAAGTGTGATATTATAACATTTCATGACTACTGCAAGACGG |  |
| Cy5-znuABC-r | CCGTCTTGCAGTAGTCATGAAATGTTATAATATCACACTTCTCATATTCGG |  |
| Cy5-pliG-f | /5Cy5/CCATGAGTACGATGAACTGTTATAATATAACAATCCCTAACGGGAGAAGG |  |
| Cy5-pliG-r | CCTTCTCCCGTTAGGGATTGTTATATTATAACAGTTCATCGTACTCATGG |  |
| Cy5-zinT-f | /5Cy5/CCCATTTTGCTATATGTTACAATATAACATTACACATCATATACATTAAGG |  |
| Cy5-zinT-r | CCTTAATGTATATGATGTGTAATGTTATATTGTAACATATAGCAAAATGGG |  |
| Cy5-L31P-f | /5Cy5/CCATCATTTTTACCTGTTATGTTATAACATAACCATAAAGATACATGCTGG |  |
| Cy5-L31P-r | CCAGCATGTATCTTTATGGTTATGTTATAACATAACAGGTAAAAATGATGG |  |
|  |  |  |

^a^ Underlined sequences indicates sequence of desired mutation

^b^ Bromination sites ordered from Integrated DNA technologies (IDT)

^c^ The annotation “/5Cy5/” corresponds to the single Cy5 fluorescent probe covalently attached to the 5′end of the DNA
