# Supplementary material for: Structural and Mechanistic Basis of Zinc Regulation Across the E. coli Zur Regulon
Source: PLoS Biol. 2014 Nov 4;12(11):e1001987. doi: 10.1371/journal.pbio.1001987 (PMC4219657; doi:10.1371/journal.pbio.1001987)
Supplement: Scheme S1 — Derivation of the stepwise microscopic equilibrium expressions. Mass-balance calculation for the stepwise binding events for (Zur2)2-DNA binding. (DOCX) [file pbio.1001987.s020.docx]

To describe the binding of Zur to DNA, we consider a stepwise model of binding equilibrium:

$Zur_{2} - DNA$ ⇌ $Zur_{2}$ + $DNA$ with $K_{d1}=\frac{[Zur_{2}][DNA]}{[Zur_{2} - DNA]}$ (S1)

$(Zur_{2})_{2} - DNA$ ⇌ $Zur_{2}$ + $Zur_{2} - DNA$ with $K_{d2}=\frac{[Zur_{2}][Zur_{2} - DNA]}{[(Zur_{2})_{2} - DNA]}$ (S2)

Subject to the mass balance:

$DNA_{total}$ = $DNA_{free}$+$Zur_{2} - DNA$ + $(Zur_{2})_{2 } - DNA$ (S3)

Rearranging S1 and S2:

$[Zur_{2} - DNA]=\frac{[Zur_{2}][DNA]}{K_{d1}}$ and $[(Zur_{2})_{2} - DNA]=\frac{[Zur_{2}][Zur_{2} - DNA]}{K_{d2}}=\frac{[Zur_{2}]^{2}[DNA]}{K_{d1}K_{d2}}$

Substitution into S3:

$$DNA_{total}=DNA_{free}+\frac{[Zur_{2}]}{K_{d1}}DNA_{free}+\frac{[Zur_{2}]^{2}}{K_{d1}K_{d2}}DNA_{free}$$

Dividing by DNA_free_ and solving for a common denominator:

$$\frac{DNA_{free}}{DNA_{total}}=\frac{K_{d1}K_{d2}}{K_{d1}K_{d2}+K_{d2}[Zur_{2}]+[Zur_{2}]^{2}}$$

Where Fraction Bound = $1-\frac{DNA_{free}}{DNA_{total}}=$ $1-\frac{K_{d1}K_{d2}}{K_{d1}K_{d2}+K_{d2}[Zur_{2}]+[Zur_{2}]^{2}}$
